# Metagenomic analysis sheds light on the mixotrophic lifestyle of bacterial phylum *Zhuqueibacterota*

**DOI:** 10.1002/imt2.249

**Published:** 2024-11-23

**Authors:** Zheng‐Han Lian, Nimaichand Salam, Sha Tan, Yang Yuan, Meng‐Meng Li, Yu‐Xian Li, Ze‐Tao Liu, Chao‐Jian Hu, Ai‐Ping Lv, Yu‐Ting OuYang, Cai‐Yu Lu, Jing‐Yi Zhang, Ying Chen, Le‐Bin Chen, Zhen‐Hao Luo, Bin Ma, Zheng‐Shuang Hua, Jian‐Yu Jiao, Wen‐Jun Li, Lan Liu

**Affiliations:** ^1^ State Key Laboratory of Biocontrol, Guangdong Provincial Key Laboratory of Plant Stress Biology and Southern Marine Science and Engineering Guangdong Laboratory (Zhuhai), School of Life Sciences Sun Yat‐Sen University Guangzhou China; ^2^ National Agri‐Food Biotechnology and Biomanufacturing Institute Mohali Punjab India; ^3^ Chinese Academy of Sciences Key Laboratory of Urban Pollutant Conversion, Department of Environmental Science and Engineering University of Science and Technology of China Hefei China; ^4^ Institute of Soil and Water Resources and Environmental Science, College of Environmental and Resource Sciences Zhejiang University Hangzhou China; ^5^ Zhejiang Provincial Key Laboratory of Agricultural Resources and Environment Zhejiang University Hangzhou China; ^6^ State Key Laboratory of Desert and Oasis Ecology, Key Laboratory of Ecological Safety and Sustainable Development in Arid Lands, Xinjiang Institute of Ecology and Geography Chinese Academy of Sciences Urumqi China

## Abstract

*Zhuqueibacterota* is a novel bacterial phylum proposed based on hot spring metagenomes and public metagenome‐assembled genomes, classified within the *Fibrobacterota*‐*Chlorobiota*‐*Bacteroidota* superphylum. This globally distributed phylum consists of one class and five orders, with the majority of its members being facultative anaerobes. Notably, the order *Zhuqueibacterales* utilizes hydrogen as an electron donor for carbon fixation through the Calvin Benson Bassham cycle. Phylogenetic and metabolic analyses reveal the phylum's key role in the carbon cycle, with frequent horizontal gene transfer events influencing its evolutionary trajectory.

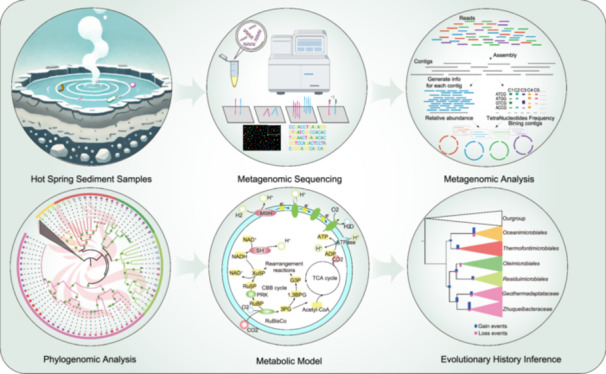

To the editor,

The candidate phylum KSB1 has been identified as anaerobic heterotrophic bacterium and detected in various environments. However, little known about its diversity, ecology, and evolutionary history. In this study, we analyzed 30 new metagenome‐assembled genomes (MAGs) from the Tengchong hot spring and 45 public MAGs. Phylogenetic analyses based on the draft genome and 16S rRNA gene indicate they form a distinct group within the *Fibrobacterota*‐*Chlorobiota*‐*Bacteroidota* (FCB) superphylum, and thus we propose their classification as a new phylum, herein *Zhuqueibacterota*, which contain five orders and one class. Members of *Zhuqueibacterota* are globally distributed and likely facultative anaerobes, with one order exhibiting autotrophy via the Calvin Benson Bassham (CBB) cycle by using hydrogen as an electron donor. Metabolic predictions at the community level suggest that *Zhuqueibacterota* play a significant role in the carbon fixation of hot spring. Ancestral state reconstruction points to frequent horizontal gene transfer (HGT) events throughout *Zhuqueibacterota* evolution. Facultative anaerobic traits appear to be ancestral, with some lineage losing hydrogen oxidation capabilities while acquiring carbon fixation capabilities through HGT in *Zhuqueibacterales*. These results shed light on diversity, ecological roles, and evolutionary history of *Zhuqueibacterota*, highlighting their significance in the carbon cycle.

## ADVANCES IN THE STUDY OF *ZHUQUEIBACTEROTA*

The earth harbors a huge biodiversity of eukaryotic and prokaryotic microorganisms. However, only a small portion of bacteria is culturable under laboratory conditions and most bacterial phyla have no cultured members. The candidate bacterial phylum KSB1, as one of the uncultured phyla, was first identified in sulfur‐rich marine sediments, and now was recognized for its broad ecological adaptability [1]. In estuary sediments, genome studies revealed its capacity for carbohydrate metabolism and *β*‐oxidation [2]. KSB1 members from hydrothermal sediment possess genes encoding benzylsuccinate synthase and alkylsuccinate synthase, suggesting a role in anaerobic hydrocarbon degradation [3]. In wetland sediments, KSB1 MAGs encode enzymes such as isopropanol dehydrogenase, phosphotransbutyrylase, and butyrate kinase, further highlighting their ecological versatility. Notably, KSB1 was also recovered from metagenomic data of hot spring, indicating KSB1 may harbor new functional niches that allow it to grow in the extreme environment. In addition, although nearly 100 MAGs of KSB1 have been reconstructed from various environments, the limited number of high‐quality genomes hinders the ability to understand the ecological roles and metabolism of KSB1. Thus, more comprehensive research is needed to explore their metabolic pathways and evolutionary history.

To address this, we collected available KSB1 genomes from public database and extracted new MAGs from hot spring samples. Our study significantly expands the genomic diversity of KSB1, clarify the phylogenetic relationships, and revealing its facultative anaerobic lifestyle and carbon fixation potential via the CBB cycle. We hereby designate this phylum as *Zhuqueibacterota*.

## DISCOVERY OF *ZHUQUEIBACTEROTA*, A CANDIDATE BACTERIAL PHYLUM IN TERRESTRIAL HOT SPRINGS

Continuous sampling of hot springs in Tengchong County, Yunnan province, China, has been conducted for metagenomic studies since 2016, and 30 high‐quality MAGs (Table S1) assigned to KSB1 were reconstructed from 16 metagenomes of hot springs. In total, 75 high‐quality KSB1 MAGs were used to construct phylogenetic trees, including 45 genomes (Table S2) downloaded from GenBank. A maximum‐likelihood tree, employing Bac120 marker sets from genome taxonomy database (GTDB), revealed that KSB1 forms a monophyletic clade within the FCB superphylum [4], and clearly separated from other phyla (Figure S1A). Phylogenetic tree based on 16S rRNA gene further supports the classification of KSB1 as a distinct phylum taxon in the domain *Bacteria* under the kingdom *Psuedomonadati* [5] (Figure S1B).

**FIGURE 1:**
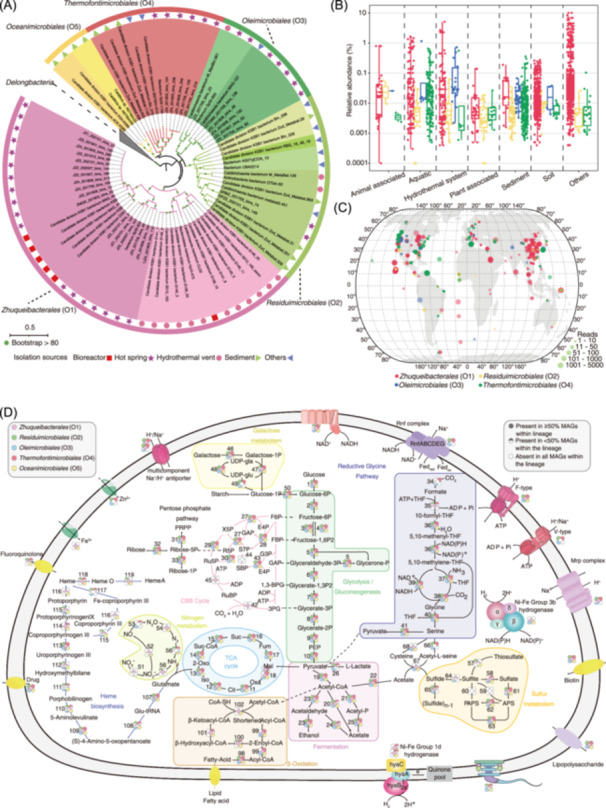
Phylogenetic placement, global distribution, and metabolic reconstruction of *Zhuqueibacterota*. (A) Maximum likelihood tree based on the concatenated alignment of 120 marker genes. Internal nodes with a bootstrap value ≥80 are indicated by solid black circles. Clade colors correspond to order‐level taxonomic classifications. (B) Relative abundances of each order of *Zhuqueibacterota* across different isolation environments. The red color represents *Zhuqueibacterales* (O1), yellow represents *Residuimicrobiales* (O2), blue represents *Oleimicrobiales* (O3), and green represents *Thermofontimicrobiales* (O4). (C) Maps showing the geographical locations of biosamples where *Zhuqueibacterota* were detected. The distribution of *Oceanimicrobiales* (O5) was not assessed due to the absence of 16S rRNA gene sequences in *Oceanimicrobiales* (O5) Metagenome‐assembled genomes (MAGs). (D) Overview of the metabolic potential of *Zhuqueibacterota*. Filled circles represent genes or metabolic pathways present in ≥50% of MAGs, half‐filled circles indicate those present in <50% of MAGs, and hollow circles represent genes or metabolic pathways absent in all MAGs. Circles are color‐coded by orders of *Zhuqueibacterota*. The numbers next to the squares correspond to the genes listed in Table S6.

To resolve the phylogenetic affiliations within phylum *Zhuqueibacterota*, we constructed a maximum likelihood tree with a concatenated set of single‐copy marker genes. Previous analyses identified four clades within KSB1 [1]; however, our phylogenetic analysis revealed five distinct groups, corresponding to five orders of the undescribed phylum KSB1 in GTDB r214 (Figure 1A) and clarified that clade II (GTDB taxonomy: p__JdFR‐76) is not part of KSB1 but a sister phylum. Average amino acid identity (AAI, 95%–100% for same species) [6], and average nucleotide identity (ANI, 95%–100% for same species) (Table S3, Figure S2) were used to classify the novel lineage on the species, which were consistent with the GTDB classification. In addition, phylogenomic tree and GTDB toolkit (GTDB‐Tk) (Tables S1 and S2) based analyses were performed to classify the novel lineage on genus levels and higher taxonomic ranks. Finally, we identified 1 class, 5 orders, 14 families, 27 genera, and 40 species from the 75 MAGs (Table S4), including 9 novel species from our own 30 MAGs. Following the recommendations for defining species and higher ranks of not‐yet‐cultured bacteria using MAGs as type material [6, 7], we proposed the name *Zhuqueibacterota* phyl. nov. for the KSB1 with five orders: *Zhuqueibacterales* (O1), *Residuimicrobiales* (O2), *Oleimicrobiales* (O3), *Thermofontimicrobiales* (O4), and *Oceanimicrobiales* (O5).

## GLOBAL DISTRIBUTION OF *ZHUQUEIBACTEROTA*

Using 16S rRNA gene sequences as queries in the IMNGS platform, *Zhuqueibacterota* was detected across six biotopes: aquatic, sediment, soil, hydrothermal vents, animal‐ and plant‐associated environments (Figure 1B). The phylum is particularly prevalent in soil and biofilm samples, where it constitutes 3%–10% of biofilm‐associated communities (Table S5), suggesting that biofilms may serve as a protective niche for these bacteria. *Zhuqueibacterales* (O1), the most widely distributed order (Figure 1C), was found in diverse environments. *Residuimicrobiales* (O2) and *Oleimicrobiales* (O3) were predominantly detected in hydrothermal vents and sediment, and *Thermofontimicrobiales* (O4) was enriched in marine environments. Despite limited data for *Oceanimicrobiales* (O5), *Zhuqueibacterota*'s broad ecological presence (Figure 1C) highlights its adaptability across various habitats, emphasizing its ecological significance.

## FACULTATIVE ANAEROBIC TRAITS OF *ZHUQUEIBACTEROTA*

Previous studies suggested that *Zhuqueibacterota* is enriched in anoxic environments [1], leading to the hypothesis of an anaerobic lifestyle. In our study, genes associated with anaerobic metabolism, such as acetyl‐CoA synthetase (*acs*), phosphate acetyltransferase (*pta*), acetate kinase (*ack*), aldehyde dehydrogenase (*aldh*), and alcohol dehydrogenase (*adh*), were detected across all clades of *Zhuqueibacterota* (Table S6), suggesting they may have the ability to produce ethanol through fermentation [8]. Additionally, we identified the Rnf complexes [9] were widely distributed among *Residuimicrobiales* (O2), *Oleimicrobiales* (O3), and *Thermofontimicrobiales* (O4). Phylogenetic analysis of the *rnfABCDEG* operon indicated that they clustered with anaerobic microorganisms (Figure S3), further suggesting the potential anaerobic lifestyle of *Residuimicrobiales* (O2), *Oleimicrobiales* (O3), and *Thermofontimicrobiales* (O4).

Remarkably, *Zhuqueibacterota* harbored *cydAB* genes, encoding the cytochrome bd ubiquinol oxidase, which provides an O_2_‐directed respiratory chain under low‐oxygen conditions. The *coxABC* genes [10] were identified in all MAGs of the *Zhuqueibacterales* (O1) and *Oceanimicrobiales* (O5), as well as in three MAGs of the *Residuimicrobiales* (O2) and two MAGs of the *Thermofontimicrobiales* (O4). Additionally, we observed that several MAGs from *Zhuqueibacterales* (O1) and *Oceanimicrobiales* (O5) encoded the aerobic aa3‐type cytochrome c oxidase [10]. However, the genes of complete pathways for oxidative phosphorylation were just widely distributed in *Zhuqueibacterales* (O1) and *Oceanimicrobiales* (O5). These findings suggest that members of *Zhuqueibacterales* (O1) and *Oceanimicrobiales* (O5) may have the ability to utilize oxygen as a terminal electron acceptor.

Considering these observations, we propose that members of *Zhuqueibacterales* (O1) and *Oceanimicrobiales* (O5) are more likely facultative anaerobes, rather than being obligate anaerobes.

## MIXOTROPHIC LIFESTYLE AND ECOLOGICAL ROLE OF *ZHUQUEIBACTEROTA* IN HOT SPRING

**FIGURE 2:**
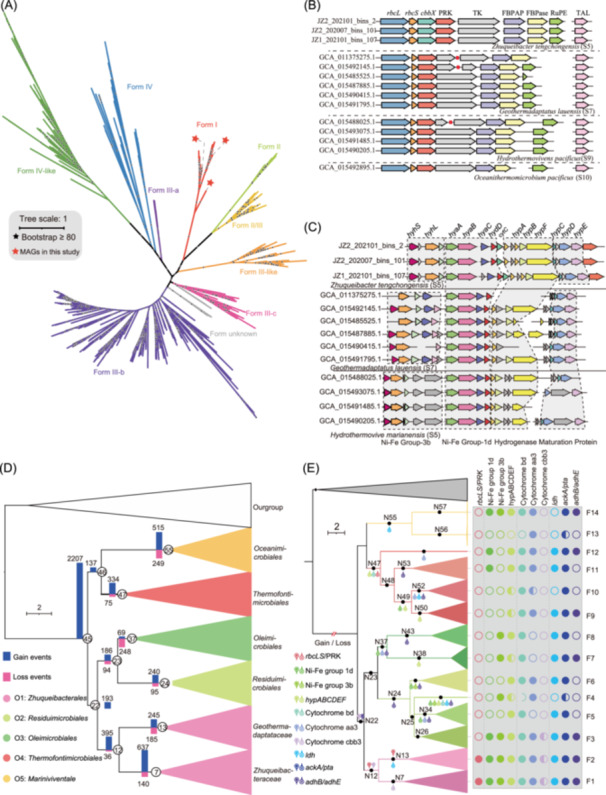
Key gene identification and evolutionary history reconstruction. (A) Phylogenetic tree of the *rbcL* gene, with internal nodes displaying a bootstrap value ≥80 marked by solid black stars. Clade colors represent different *rbcL* forms. (B) Gene cluster organization is associated with the Calvin–Benson–Bassham (CBB) cycle. (C) Organization of the hydrogenase gene cluster. (D) Ancestral genome content reconstruction was conducted using COUNT software, based on the Bayesian tree from MyBayes. Histograms next to internal nodes indicate gene gain (blue) and loss (pink) events. (E) Inferred gene gain and loss events pertaining to the CBB cycle, hydrogenase, and terminal oxidase in *Zhuqueibacterota*.

Through metabolic reconstruction, all *Zhuqueibacterota* orders contain complete genes for glycolysis, gluconeogenesis, and pentose phosphate pathways (Figure 1D). MAGs assigned to the *Zhuqueibacterales* (O1), *Thermofontimicrobiales* (O4), and *Oceanimicrobiales* (O5) harbor the complete gene sets for the TCA cycle. We also identified a diverse array of carbohydrate‐active enzymes (CAZymes) among the 75 MAGs, including 79 glycoside hydrolases (GHs), 10 glycosyl transferases (GTs), 10 carbohydrate esterases (CEs), 8 polysaccharide lyases (PLs), and 10 carbohydrate‐binding modules (CBMs) (Table S7). Interestingly, the gene encoding the large subunit of ribulose‐1,5‐bisphosphate carboxylase/oxygenase (RuBisCO) was identified in 35 MAGs of *Zhuqueibacterales* (O1). Phylogenetic analysis revealed that the RuBisCO large subunit (*rbcL*) gene belongs to form I group (Figure 2A), which plays a crucial role in carbon fixation via the CBB cycle [11]. Moreover, genes encoding RuBisCO small subunit (*rbcS*), phosphoribulokinase (PRK), transketolase (TKT), fructose‐1,6‐bisphosphate aldolase (FBPA), fructose‐1,6‐bisphosphatase (FBPase), and ribulose‐5‐phosphate 3‐epimerase (RuPE) were found near the *rbcL* gene cluster (Figure 2B). Based on these observations, we hypothesize that members of *Zhuqueibacterota* possess the capability for carbon fixation via the CBB cycle. Notably, the absence of sedoheptulose‐1,7‐bisphosphatase (SBPase) and the presence of transaldolase (TAL) suggest that *Zhuqueibacterota* uses the transaldolase‐variant of the CBB cycle [12, 13].

Carbon fixation via the CBB cycle requires significant energy and reducing equivalents, and hydrogen is a critical energy source in terrestrial hot springs [14] and hydrothermal vents [15]. Numerous prokaryotes can utilize H_2_ and CO_2_ as their energy and carbon source [16]. In our study, genes annotated to encode hydrogenases were present in *Zhuqueibacterota*, and the phylogenetic analyses of hydrogenases revealed five different clades ([Ni–Fe] group 1d, 3b‐3d, and [Fe–Fe] group) (Figure S4). In *Zhuqueibacterales* (O1) MAGs, especially those containing CBB gene cluster, we identified two types of hydrogenases (Figure 2C). The [Ni–Fe]‐group 1d hydrogenase, a membrane‐bound hydrogenase, transfers electrons from H_2_ to the respiratory chain via membrane‐connected cytochromes [17] (Figure S4). Additionally, the cytosolic [Ni–Fe]‐group 3b hydrogenase transfers electrons generated by H_2_ oxidation to NAD^+^ [18], which serves as a reducing equivalent, for carbon fixation or for ATP production through the respiratory chain [17]. The biosynthesis and maturation of [Ni–Fe] hydrogenase are catalyzed by six Hyp proteins (*hypABCDEF*) [19]. The co‐localization of hydrogenase maturation proteins, [Ni–Fe]‐group 1 d and 3b hydrogenases (Figure 2C) indicate that *Zhuqueibacterales* (O1) likely utilizes hydrogen as an energy source for carbon fixation.

The CBB cycle operates aerobically [20], and both the [Ni–Fe]‐group 1d and 3b hydrogenases exhibit O_2_ tolerance, maintaining hydrogen oxidation even in the presence of oxygen. This further supports the hypothesis that *Zhuqueibacterota* is facultative anaerobic mixotrophy.

Utilizing METABOLIC v4.0, we constructed a community metabolic network comprising with 186 medium/high‐quality MAGs (completeness >50% and contamination <6%) derived from JZ2 hot spring (JZ2_202007). Despite being represented by only two MAGs, *Zhuqueibacterota* exhibited the highest relative abundance (15.48%). It contributed to 24 metabolic processes, with a relative contribution exceeding 25% (Figure S5, Table S8). Notably, *Zhuqueibacterota* dominated carbon fixation via the CBB cycle, with the highest contribution (57.9%) among all microbial taxa (Table S8). These findings suggest that *Zhuqueibacterota*, with its chemolithoautotrophic capabilities, plays a vital role in this ecosystem.

## THE EVOLUTIONARY HISTORY OF *ZHUQUEIBACTEROTA*

To decipher the evolutionary histories of the *Zhuqueibacterota*, we predicted gene gain and loss events mapping onto a Bayesian tree showed that contemporary *Zhuqueibacterota* harbor more gene families over time (Figure 2D, Figure S6), with 71% of HGT events occurred within *Zhuqueibacterales* (O1), mostly from hot spring‐associated MAGs. HGT played a major role in genome diversity, despite the common trend of genome reduction in hot spring environments. The ancestral *Zhuqueibacterota* likely possessed hydrogenase, with later loss events leading to the loss of this hydrogen utilization capability in some lineages. The *cydAB* gene (Figure 2E, Table S9) for high oxygen stress response was acquired early, while the *coxABC* gene was independently acquired by different lineages (Figure 2E, Table S9). Anaerobic fermentation‐related genes were lost at multiple evolutionary nodes, potentially contributing to metabolic diversity, indicating that the ancestors of *Zhuqueibacterota* were facultative anaerobic bacteria. Genes related to the CBB cycle, such as *rbcL*, *rbcS*, and PRK, were acquired gradually. Over time, *Zhuqueibacterota* inhabiting thermal environments gained the ability for carbon fixation via the CBB cycle.

## CONCLUSION

In this study, we have expanded our understanding of the uncultured phylum KSB1, herein *Zhuqueibacterota*, by utilizing high‐quality genomes to explore its diversity and function. Our analysis indicated that *Zhuqueibacterota* has clear functional differentiation, and distinct environmental preferences among its clades. Notably, this phylum represents a group of facultative anaerobic chemoautotrophs, which play a pivotal role in sustaining microbial communities in hot springs. Ancestral state reconstructions suggest the facultative anaerobic lifestyle of their ancestor, while carbon fixation capabilities probably evolved through HGT. Overall, this study provides new insights into the ecological functions of *Zhuqueibacterota* and open avenues for further research. Moreover, the findings indicate the possibility of isolating and purifying members of this phylum using the hydrogen‐oxidizing autotrophic enrichment system.

## AUTHOR CONTRIBUTIONS

**Zheng‐Han Lian**: Writing—review and editing; writing—original draft; software. **Nimaichand Salam**: Writing—review and editing; formal analysis; data curation; investigation. **Sha Tan**: Writing—review and editing; software; visualization. **Yang Yuan**: Writing—review and editing; software; visualization. **Meng‐Meng Li**: Writing—review and editing; software; resources. **Yu‐Xian Li**: Writing—review and editing; visualization; software. **Ze‐Tao Liu**: Writing—review and editing; data curation. **Chao‐Jian Hu**: Writing—review and editing; methodology. **Ai‐Ping Lv**: Writing—review and editing; formal analysis. **Yu‐Ting OuYang**: Writing—review and editing; validation. **Cai‐Yu Lu**: Writing—review and editing; formal analysis. **Jing‐Yi Zhang**: Writing—review and editing; visualization. **Ying Chen**: Writing—review and editing; software; data curation. **Le‐Bin Chen**: Writing—review and editing; software; resources. **Zhen‐Hao Luo**: Writing—review and editing; resources; validation. **Bin Ma**: Writing—review and editing; visualization; software. **Zheng‐Shuang Hua**: Writing—review and editing; software. **Jian‐Yu Jiao**: Supervision; writing—review and editing; writing—original draft; software; funding acquisition. **Wen‐Jun Li**: Supervision; writing—review and editing; funding acquisition; project administration; resources. **Lan Liu**: Supervision; writing—original draft; writing—review and editing; visualization; software; validation; funding acquisition; project administration; data curation; resources.

## CONFLICT OF INTEREST STATEMENT

The authors declare no conflicts of interest.

## ETHICS STATEMENT

No animals or humans were involved in this study.

## Supporting information


**Figure S1.** Phylogeny of *Zhuqueibacterota*.
**Figure S2.** The ANI/AAI heatmap of all pairwise comparisons.
**Figure S3.** The phylogenetic tree based on concatenated alignment of Rnf complex genes.
**Figure S4.** Phylogenetic trees and classification of hydrogenases and metabolic model of hydrogen‐oxidizing bacteria.
**Figure S5.** Microbial contribution to carbon, nitrogen, and sulfur cycling in hot spring.
**Figure S6.** Ancestral genome content reconstruction using COUNT software.
**Figure S7.** Phylogenetic tree of *nosZ* gene.
**Figure S8.** Variation and correlation of genome size and GC content across orders of *Zhuqueibacterota*.
**Figure S9.** Plot of Principal Coordinates Analysis (PCoA) based on functional traits of *Zhuqueibacterota* MAGs.

**Table S1.** General genomic features of Candidate Division KSB1 genomes from hot spring.
**Table S2.** Genomic overview of Candidate Division KSB1 reference genome.
**Table S3.** ANI/AAI values for each genome pair.
**Table S4.** Brief nomenclature of *Zhuqueibacterota* MAGs.
**Table S5.** Global distribution and relative abundances of the *Zhuqueibacterota*.
**Table S6.** List of genes in the metabolic model of *Zhuqueibacterota*.
**Table S7.** CAZyme statistics for *Zhuqueibacterota* MAGs.
**Table S8.** The MW‐score for JZ2 hot spring metagenomic dataset.
**Table S9.** Gene gain and loss events detected at key nodes.

## Data Availability

The MAGs discussed in this research have been archived in the GenBank database under the BioProject ID PRJNA895542 (https://www.ncbi.nlm.nih.gov/bioproject/PRJNA895542/). Accession numbers for each individual MAG are detailed in Table S1. The data and scripts used are saved in GitHub https://github.com/lianzhh-pub/Code_for_iMeta2024. Supplementary materials (methods, figures, tables, graphical abstract, slides, videos, Chinese translated version, and updated materials) may be found in the online DOI or iMeta Science http://www.imeta.science/. The data that supports the findings of this study are available in the supplementary material of this article.
