# Supplementary material for: Metagenomic analysis sheds light on the mixotrophic lifestyle of bacterial phylum Zhuqueibacterota
Source: Imeta. 2024 Nov 23;3(6):e249. doi: 10.1002/imt2.249 (PMC11683472; doi:10.1002/imt2.249)
Supplement: Supplementary file 1 — Figure S1. Phylogeny of Zhuqueibacterota. Figure S2. The ANI/AAI heatmap of all pairwise comparisons. Figure S3. The phylogenetic tree based on concatenated alignment of Rnf complex genes. Figure S4. Phylogenetic trees and classification of hydrogenases and metabolic model of hydrogen‐oxidizing bacteria. Figure S5. Microbial contribution to carbon, nitrogen, and sulfur cycling in hot spring. Figure S6. Ancestral genome content reconstruction using COUNT software. Figure S7. Phylogenetic tree of nosZ gene. Figure S8. Variation and correlation of genome size and GC content across orders of Zhuqueibacterota. Figure S9. Plot of Principal Coordinates Analysis (PCoA) based on functional traits of Zhuqueibacterota MAGs. [file IMT2-3-e249-s002.docx]

# **Supporting information to**

# Metagenomic analysis sheds light on the mixotrophic lifestyle of bacterial phylum *Zhuqueibacterota*

**Running title:** Mixotrophic lifestyle of *Zhuqueibacterota*

Zheng-Han Lian^1^, Nimaichand Salam^2^, Sha Tan^1^, Yang Yuan^1^, Meng-Meng Li^1^, Yu-Xian Li^3^, Ze-Tao Liu^1^, Chao-Jian Hu^1^, Ai-Ping Lv^1^, Yu-Ting OuYang^1^, Cai-Yu Lu^4,5^, Jing-Yi Zhang^1^, Ying Chen^1^, Le-Bin Chen^1^, Zhen-Hao Luo^1^, Bin Ma^4,5^, Zheng-Shuang Hua^3^, Jian-Yu Jiao^1*^, Wen-Jun Li^1,6*^, Lan Liu^1*^

^1^State Key Laboratory of Biocontrol, Guangdong Provincial Key Laboratory of Plant Stress Biology and Southern Marine Science and Engineering Guangdong Laboratory (Zhuhai), School of Life Sciences, Sun Yat-Sen University, Guangzhou, 510275, China

^2^National Agri-Food Biotechnology and Biomanufacturing Institute, Sector-81 (Knowledge City), Mohali, 140306, Punjab, India

^3^Chinese Academy of Sciences Key Laboratory of Urban Pollutant Conversion, Department of Environmental Science and Engineering, University of Science and Technology of China, Hefei 230026, China

^4^Institute of Soil and Water Resources and Environmental Science, College of Environmental and Resource Sciences, Zhejiang University, Hangzhou 310058, China

^5^Zhejiang Provincial Key Laboratory of Agricultural Resources and Environment, Zhejiang University, Hangzhou 310058, China

^6^State Key Laboratory of Desert and Oasis Ecology, Key Laboratory of Ecological Safety and Sustainable Development in Arid Lands, Xinjiang Institute of Ecology and Geography, Chinese Academy of Sciences, Urumqi, 830011, China

**Correspondence:** [liulan9@mail.sysu.edu.cn](mailto:liulan9@mail.sysu.edu.cn) (Lan Liu); [liwenjun3@mail.sysu.edu.cn](mailto:liwenjun3@mail.sysu.edu.cn) (Wen-Jun Li); [jiaojy5@mail.sysu.edu.cn](mailto:jiaojy5@mail.sysu.edu.cn) (Jian-Yu Jiao)

TABLE OF CONTENTS

[SUPPLEMENTARY TEXT 3](#_Toc179973884)

[Method details 3](#_Toc179973885)

[Supplementary Results 6](#_Toc179973886)

[Nomenclature of *Zhuqueibacterota* phylum members 7](#_Toc179973887)

[SUPPLEMENTARY FIGURES 24](#_Toc179973888)

[Figure S1. Phylogeny of *Zhuqueibacterota.* 24](#_Toc179973889)

[Figure S2. The ANI/AAI heatmap of all pairwise comparisons. 25](#_Toc179973890)

[Figure S3. The phylogenetic tree based on concatenated alignment of Rnf complex genes. 26](#_Toc179973891)

[Figure S4. Phylogenetic trees and classification of hydrogenases and metabolic model of hydrogen-oxidizing bacteria. 27](#_Toc179973892)

[Figure S5. Microbial contribution to carbon, nitrogen, and sulfur cycling in hot spring. 28](#_Toc179973893)

[Figure S6. Ancestral genome content reconstruction using COUNT software. 30](#_Toc179973894)

[Figure S7. Phylogenetic tree of *nosZ* gene. 31](#_Toc179973895)

[Figure S8. Variation and correlation of genome size and GC content across orders of *Zhuqueibacterota*. 32](#_Toc179973896)

[Figure S9. Plot of Principal Coordinates Analysis (PCoA) based on functional traits of *Zhuqueibacterota* MAGs. 33](#_Toc179973897)

[Reference 34](#_Toc179973898)

## SUPPLEMENTARY TEXT

### **Method details**

**Sample collection, DNA extraction, and metagenomic sequencing**

A total of 16 sediment samples were collected from hot springs in Tengchong, Yunnan, China, using sterile spoons. The samples were immediately frozen in liquid nitrogen and stored at -80°C until DNA extraction. In situ temperature and pH were measured with a temperature/pH probe (DR850, HACH Company, CO, USA). DNA was extracted from approximately 20 g of sediment using the PowerSoil DNA Isolation Kit (MoBio, CA, USA), following the manufacturer's protocols [1]. The concentration of extracted DNA was quantified using a Qubit fluorometer. Metagenomic libraries with an average insert size of 350 bp were then prepared and sequenced on the Illumina HiSeq X Ten platform at Azenta, Suzhou, China. This sequencing effort generated approximately 650 Gbp of paired-end reads (2 × 150 bp).

**Metagenome assembly and genome binning**

Raw reads underwent quality control as previously described [2], involving four filtering steps. First, adapter sequences were removed, followed by the elimination of PCR duplicate reads. Reads containing more than 10% ambiguous bases or exhibiting low complexity were discarded. Finally, reads with quality scores below 30 at the 3' end were trimmed. After filtering, metagenome assembly was performed using SPAdes (v3.15.5) [3] with the parameters '--meta -k 21,33,55,77,99,127'. Scaffolds longer than 2500 bp were selected for further analysis. Coverage for each assembly was estimated by mapping high-quality reads using BBMap (version v36.x; <https://sourceforge.net/projects/bbmap/>) with the parameters 'minid = 0.9 k = 15'. Genome binning of the assembled scaffolds was conducted with MetaBAT2 [4], utilizing coverage data and tetranucleotide frequency (TNF). The quality of each genome bin was assessed for completeness, contamination, and heterogeneity using CheckM (version 1.0.12) [5]. To optimize the genome bins, clean reads mapped by BBMap were reassembled using SPAdes (v3.15.5) with the options '--careful -k 21,33,55,77,99,127'. Genome bins with at least 80% completeness and no more than 5% contamination were retained for further analysis.

**Functional annotation of genomes**

For metabolic function analyses, genomes from both this study and public databases were used for functional annotation. A total of 45 publicly available genomes were manually selected and downloaded from the NCBI database (details in Table S2). Open reading frames (ORFs) were predicted using Prodigal (v2.6.3, default parameters) [6], and functional annotation of the genes was conducted by querying them against several databases, including NCBI-nr, eggNOG, and KEGG. Functional annotation using the eggNOG database (v5.0) was performed with eggNOG-mapper (v2.1.8) [7]. KEGG pathway analysis was carried out independently using both the KEGG Automatic Annotation Server (KAAS) [8] and KofamScan (v1.3.0) [9]. Additionally, tRNAs and rRNAs were identified using Aragorn (v1.2.38) [10] and Barrnap (v0.9) (https://github.com/tseemann/barrnap.git), respectively. Carbohydrate-active enzymes (CAZymes) were identified using a local implementation of dbCAN2 (v3.0.2) [11], with annotations retained only if confirmed by all three tools: HMMER, DIAMOND, and eCAMI.

**Phylogenetic analysis**

Phylogenomic tree

The taxonomic classification of the metagenome-assembled genomes (MAGs) was performed using GTDB-Tk (v2.1.0) [12], confirming all genomes belong to the KSB1 phylum. For downstream analyses, only high-quality MAGs with a minimum completeness of 80% and contamination below 5%, as estimated by CheckM [5], were retained. A total of 75 genomes were utilized to construct phylogenomic trees. The Bac120 marker set was applied to determine the phylogenetic placement of the KSB1 (*Zhuqueibacterota*) MAGs, with marker proteins identified using GTDB-Tk [12]. Maximum-likelihood trees were constructed using IQ-TREE (v1.6.10) [13], with parameters (-alrt 100 -bb 1000 -nt AUTO). The best-fit substitution model (LG + R5) was selected using ModelFinder [14], supported by the Akaike Information Criterion (AIC), corrected AIC, and Bayesian Information Criterion (BIC).

16S rRNA gene tree

To infer the 16S rRNA gene phylogeny, sequences from the MAGs in this study and reference sequences from the SINTAX database (https://www.drive5.com/sintax/rdp_16s_v18.fa.gz) were clustered at 97% sequence similarity. Representative sequences were selected for further analysis. The sequences were aligned using the super5 algorithm in MUSCLE5 [15], and poorly aligned regions were trimmed using trimAl (v1.4. rev22) [16]. A maximum-likelihood tree was constructed using IQ-TREE [13].

*rbcL* gene tree

Reference sequences of RuBisCo large subunit (*rbcL*) were obtained from prior studies [17]. The *rbcL* genes of the MAGs in this study were identified using BLAST and Hmmer. Multiple sequence alignments were performed using MUSCLE [18], followed by trimming poorly aligned regions using trimAl [16]. A maximum-likelihood tree was then constructed using IQ-TREE [13].

Hydrogenase gene tree

Hydrogenase protein sequences were sourced from Greening et al. [19] and Carnevali et al. [20], with additional verification through HydDB [21]. The protein sequences from different hydrogenase groups were aligned separately using MUSCLE, and poorly aligned regions were removed with trimAl [16]. The phylogenetic tree was generated using IQ-TREE (v1.6.10) [13] with the best model parameters determined by internal selection.

*rnf* gene tree

Reference genomes for the *rnf* genes were obtained from previous studies [22] and downloaded from the NCBI GenBank database. Open reading frames (ORFs) were predicted using Prodigal [6], and functional annotation was performed using KofamScan [9]. The identified rnf gene clusters (*rnfABCDEG*) were aligned using MUSCLE [18], and poorly aligned regions were trimmed with trimAl [16]. Phylogenomic trees were constructed from concatenated alignments using IQ-TREE [16].

All phylogenetic trees, generated in Newick format, were visualized and annotated using iTOL [23].

**Global distribution and relative abundance survey**

To investigate the global distribution and abundance of KSB1 (*Zhuqueibacterota*), 16S rRNA gene sequences from the MAGs were submitted to the IMNGS webserver [24] for comparison against the Sequence Read Archive (SRA) database. Sequences with > 97% identity to the query sequences were retrieved, along with associated SRA accession numbers, sample sizes, and descriptions. A minimum overlap of 200 bp was required to ensure the quality of matched sequences. Longitude and latitude data were extracted from NCBI using the SRA accession numbers, then manually verified and corrected for accuracy. The distribution and relative abundance of *Zhuqueibacterota* were expressed as sequence hit counts per amplicon dataset. Only query hits with a count ≥ 2 were considered positive.

**Gene content comparison**

Average nucleotide identity (ANI) and average amino acid identity (AAI) were calculated using OrthoANI [25] and EzAAI [26], respectively. Orthologous sequences were inferred using OrthoFinder v2.5.5 [27]. To explore the evolutionary history of this phylum, ancestral genome content was reconstructed using the COUNT software [28] with the Dollo parsimony method. Bayesian tree topology was constructed with MrBayes [29], using the following parameters: ngen=1,000,000, Nruns=2, Nchains=4, diagnfreq=1,000, relburnin=yes, burninfrac=0.25, samplefreq=100, and printfreq=100. Candidate horizontal gene transfer (HGT) events were identified using the AvP [30] pipeline in combination with the UniRef90 database[31], which detects potential HGTs within a phylogenetic framework. Only genes labeled as HGT or HGT-NT, with a local HGT score of ≥0.8, were considered putative HGT genes.

**Function annotation of microbial community**

All MAGs of JZ2 (JZ2-202007) sample were recovered following the methodology described in the preceding section ‘Metagenome assembly and genome binning’. Taxonomic classification of these MAGs was performed using GTDB-Tk [12]. Subsequently, the metabolic potential of these MAGs was assessed using METABOLIC-C v4.0 [32] pipeline. Additionally, a community-scale network was constructed to evaluate microbial contributions to carbon, nitrogen, and sulfur cycling in hot springs by assigning metabolic weight score (MW-score) to microbial taxa.

### **Supplementary Results**

**The nitrogen metabolism and sulfur metabolism of *Zhuqueibacterota***

The *nosZ* gene, which is crucial for the final step of denitrification leading to nitrogen gas production, was detected in over 50% of metagenome-assembled genomes (MAGs) belonging to the *Zhuqueibacterales* (O1) and *Oceanimicrobiales* (O5) lineages. Nevertheless, other associated denitrification genes, such as *nirK/S* or *norBC*, were absent, prompting phylogenetic analysis that indicates *nosZ* may have been acquired via horizontal gene transfer (Figure S7). Additionally, the sporadic co-occurrences of *narGHI* and *nirB* genes in MAGs from bioreactor and hydrothermal vent environments suggest that *Zhuqueibacterales* (O1) may play a role in dissimilatory nitrate reduction within these ecosystems. Genes related to sulfur metabolism, including *sat* (sulfate adenylyltransferase), *phsA* (polysulfide reductase chain A), and *cysK* (cysteine synthase), were identified in *Zhuqueibacterota* MAGs. Notably, *sat* and *phsA* were found in nearly all MAGs, with the exception of *Oleimicrobiales* (O3), which housed only one MAG containing these genes. *sat* facilitates the transformation of inorganic sulfate to adenylyl sulfate, while *phsA* primarily functions in converting thiosulfate to sulfide, subsequently utilized by *cysK* for cysteine synthesis. The widespread presence of *cysK* across all lineages, except *Oceanimicrobiales* (O5), implies that most *Zhuqueibacterota* lineages likely contribute to assimilatory sulfate reduction.

**The comparison between orders of *Zhuqueibacterota***

Genome sizes in *Zhuqueibacterota* vary significantly, ranging from 2.19 to 6.01 Mbp (Fig. S8A, Table S1), with larger genomes generally associated with broader metabolic capacity and ecological adaptability, contributing to their presence in diverse environments [33, 34]. Bacteria with higher GC content tend to have relatively larger genomes [35]. In marine and thermophilic communities, both genome size and GC content decline in parallel, consistent with genomic streamlining [36]. Significant differences in GC content (Figure S8B) are observed among different orders, albeit with varying degrees of negative correlation between genome size and GC content within each order (Figure S8C and S8D). Given the high diversity in isolation sources, the roles of *Zhuqueibacterota* bacteria may exhibit notable differentiation. Ancestral genome content reconstruction reveals that contemporary Z*uqueibacterota* harbor more gene families (Figure S6), and HGT events are frequently observed in hot spring-associated MAGs, potentially great contributing to functional differentiation of *Zhuqueibacterota*. Principal Coordinates Analysis (PCoA) based on KEGG Orthology (KO) revealed distinct functional differences (R^2^_Adonis_ = 0.11638, P_Adonis_ = 0.002, Fig. S9) between clades within *Zhuqueibacterota*, particularly between *Zhuqueibacteraceae* (F1) and *Geothermadaptataceae* (F2). *Oceanimicrobiales* (O5) appeared to act as "transitional groups" between these two clades. *Zhuqueibacteraceae* (F1) predominantly found in terrestrial hot springs, *Geothermadaptataceae* (F2) in hydrothermal vents, and *Oceanimicrobiales* (O5) in marine sediment, with the divergence in their habitats likely driving the observed functional differences.

### **Nomenclature of *Zhuqueibacterota* phylum members**

***Zhuqueibacterota* phy. nov. (P1)**

*Zhuqueibacterota* (Zhu.quei.bac.te.ro’ta. N.L. neut. n. *Zhuqueibacter* type genus of the phylum; N.L. neut. pl. n. suff. -*ota* ending to denote a phylum; N.L. neut. pl. n. *Zhuqueibacterota* the *Zhuqueibacter* phylum.

The properties of the taxon are similar to that of the class *Zhuqueibacteria*.

Type genus: *Zhuqueibacter*.

***Zhuqueibacteria* classis nov. (C1)**

*Zhuqueibacteria* (Zhu.quei.bac.te’ri.a. N.L. neut. n. *Zhuqueibacter* type genus of the type order of the class; L. suff. -*ia* ending to denote a class; N.L. neut. pl. n. *Zhuqueibacteria* the class of the order *Zhuqueibacterales*).

The class *Zhuqueibacteria* contains the order *Zhuqueibacterales* ord. nov., *Residuimicrobiales* ord. nov., *Oleimicrobiales* ord. nov., *Thermofontimicrobiales* ord. nov. and *Oceanimicrobiales* ord. nov.

Type order: *Zhuqueibacterales*.

***Zhuqueibacterales* ord. nov. (O1)**

*Zhuqueibacterales* (Zhu.quei.bac.tera’les. N.L. masc. n. *Zhuqueibacter* type genus of the order; L. suff. -*ales* ending to denote an order; N.L. fem. pl. n. *Zhuqueibacterales* the *Zhuqueibacter* order).

The order *Zhuqueibacterales* comprised of two families *Zhuqueibacteraceae* fam. nov. and *Geothermadaptataceae* fam. nov. that are associated with hot springs and hydrothermal vent.

Type genus: *Zhuqueibacter*.

***Zhuqueibacteraceae* fam. nov. (F1)**

*Zhuqueibacteraceae* (Zhu.quei.bac.tera’ce.ae. N.L. masc. n. *Zhuqueibacter* type genus of the family; L. suff. -*aceae* ending to denote a family; N.L. fem. pl. n. *Zhuqueibacteraceae* the *Zhuqueibacter* family).

The family *Zhuqueibacteraceae* at present contains two genera *Coneutiohabitans* gen. nov. and *Zhuqueibacter* gen. nov.

Type genus: *Zhuqueibacter*.

***Coneutiohabitans*** **gen. nov.** **(G1)**

*Coneutiohabitans* (Co.ne.ut.i.o.ha’bi.tans. Gr. n. *coneutis* digester (bioreactor); L. pres. part. *habitans* inhabiting; N.L. masc. n. *Coneutiohabitans* an inhabitant of bioreactor).

Type species: *Coneutiohabitans beijingensis.*

***Coneutiohabitans thermophilus* sp. nov. (S1)**

*Coneutiohabitans thermophilus* (ther.mo’phi.lus. Gr. fem. adj. *thermê* heat; N.L. masc. adj. suff. -*philus* friend, loving; N.L. masc. adj. *thermophilus* heat-loving, reflecting the source of the genome bin as a hot spring sample).

Type material: ZMQR_201803_bins_22 (JAPDQY000000000), obtained from the metagenome assembly of a sample collected from a Tengchong hot spring in south-west China.

***Coneutiohabitans nanjingensis* sp. nov. (S2)**

*Coneutiohabitans nanjingensis* (nan.jing.en’sis. N.L. masc/fem. adj. *nanjingensis* of Nanjing, referring to the source of the genome).

Type material: MAG_40 (GCA_013359425.1), obtained from the metagenome assembly of a sample collected from bioreactor located in Nanjing, the city of PR China.

***Coneutiohabitans beijingensis* sp. nov. (S3)**

*Coneutiohabitans beijingensis* (bei.jing.en’sis. N.L. masc/fem. adj. *beijingensis* of Beijing, referring to the source of the genome).

Type material: KSB (GCA_003576975.1), obtained from the metagenome assembly of a sample collected from bioreactor located in Beijing, the capital city of PR China.

***Zhuqueibacter*** **gen. nov. (G2)**

*Zhuqueibacter* (Zhu.quei.bac’ter N.L. n. *Zhuque*, one of Four Symbols in ancient Chinese mythology, referring the geographical location of the bacteria; N.L. masc. n. *bacter*, bacterium; N.L. neut. n. *Zhuqueibacter* a microbe from hot spring**).**

Type species: *Zhuqueibacter tengchongensis.*

***Zhuqueibacter sedimenti* sp. nov. (S4)**

*Zhuqueibacter sedimenti* (se.di.men’ti. L. gen. neut. n. *sedimenti*, of sediment).

Type material: SpSt-324 (GCA_011047125.1), obtained from the metagenome assembly of a sample of hot spring sediment located at British Columbia, Canada).

***Zhuqueibacter tengchongensis* sp. nov. (S5)**

*Zhuqueibacter tengchongensis* (teng.chong.en’sis. N.L. masc./fem. adj. *tengchongensis* referring to Tengchong)

Type material: JZ2_202007_bins_101 (JAPDQB000000000), obtained from the metagenome assembly of a sample of hot spring sediment in Tengchong, PR China).

***Zhuqueibacter pondensis* sp. nov. (S6)**

*Zhuqueibacter pondensis* (pon.den’sis. N.L. masc./fem. adj. *pondensis*, referring to pond).

Type material: JZ4_201709_bins_45 (JAPDQK000000000), obtained from the metagenome assembly of a sample of sediment from an artificial hot spring reservoir in Tengchong, Yunnan, China).

***Geothermadaptataceae* fam. nov. (F2)**

*Geothermadaptataceae* (Ge.o.therm.ad.ap.ta.ta.ce’ae. N.L. masc. n. *Geothermadaptatus*, type genus of the family; L. suff. -*aceae* ending to denote a family; N.L. fem. pl. n. *Geothermadaptataceae* the *Geothermadaptatus* family).

The family *Geothermadaptataceae* at present contains four genera *Geothermadaptatus* gen. nov., *Bioreactorimicrobium* gen. nov., *Hydrothermoviven* gen. nov. and *Oceanithermomicrobium* gen. nov.

Type genus: *Geothermadaptatus*.

***Geothermadaptatus* gen. nov. (G3)**

*Geothermadaptatus* (Ge.o.therm.ad.ap.ta.tus. Gr. fem. n. *gê* the earth; Gr. masc. adj. *thermos* hot; L. masc. perf. part. *adaptatus* adapted to; N.L. masc. n. *Geothermadaptatus* adapted to (living) in hot earth).

Type species: *Geothermadaptatus lauensis.*

***Geothermadaptatus lauensis* sp. nov. (S7)**

*Geothermadaptatus lauensis* (lau.en’sis. N.L. masc. adj. *lauensis* pertaining to Lau, referring to the deep-sea hydrothermal vent in the Lau basin).

Type material: HyVt-473 (GCA_011375275.1), obtained from the metagenome assembly of a sample of hydrothermal vents in Lau Basin, Tonga.

***Bioreactorimicrobium* gen. nov. (G4)**

*Bioreactorimicrobium* (Bi.o.re.ac.to.ri.mi.cro’bi.um. N.L. neut. n. *microbium* a microbe; N.L. neut. n. *Bioreactorimicrobium* a microbe from a bioreactor).

Type species: *Bioreactorimicrobium primus*.

***Bioreactorimicrobium primus* sp. nov. (S8)**

*Bioreactorimicrobium primus* (pri’mus. L. neut. adj. *primus* first, to represent the first genome bin from of the genus).

Type material: CLD2 (GCA_008501765.1), obtained from the metagenome assembly of a sample collected from bioreactor located in KAUST, Saudi Arabia).

***Hydrothermovivens* gen. nov. (G5)**

*Hydrothermovivens* (Hy.dro.ther.mo.vi’vens. Gr. neut n. *hydôr* water; Gr. masc. adj. *thermos* heat; L. pres. part. *vivens* living; N.L. masc. n. *Hydrothermovivens* living in a hot water environment).

Type species: *Hydrothermovivens marianensis*.

***Hydrothermovivens pacificum* sp. nov. (S9)**

*Hydrothermovivens pacificum* (pa.ci’fi.cum. N.L. masc./fem. adj. *pacificum*, pertaining to the Pacific Ocean, the source of the genome).

Type material: S139_15 (GCA_015485555.1), obtained from the metagenome assembly of a hydrothermal vent sample from the Pacific Ocean.

***Oceanithermomicrobium* gen. nov. (G6)**

*Oceanithermomicrobium* (O.ce.a.ni.ther.mo.mi.cro’bi.um. L. masc. n. *oceanus* the ocean; Gr. masc. adj. *thermos* heat; N.L. neut. n. *microbium* a microbe; N.L. neut. n. *Oceanithermomicrobium* a microbe from the hot ocean (hydrothermal vent) environment).

Type species: *Oceanithermomicrobium pacificum.*

***Oceanithermomicrobium pacificum* sp. nov. (S10)**

*Oceanithermomicrobium pacificum* (pa.ci’fi.cum. L. neut. adj. *pacificum* pertaining to the Pacific Ocean, the source of the genome).

Type material: S146_25 (GCA_015492895.1), obtained from the metagenome assembly of a hydrothermal vent from Pacific Ocean.

***Residuimicrobiales* ord. nov. (O2)**

Residuimicrobiales (Re.si.du.i.mi.cro.bi.a’les. N.L. neut. n. *Residuimicrobium* type genus of the order; L. suff. -*ales* ending to denote an order; N.L. fem. pl. n. *Residuimicrobiales* the *Residuimicrobium* order).

The order *Residuimicrobiales* comprised of four families *Residuimicrobiaceae* fam. nov., *Krinioviventaceae* fam. nov. and *Sediminimicrobiaceae* fam. nov. and *Oceaniaggregativiventaceae* fam. nov.

Type genus: *Residuimicrobium*.

***Krinioviventaceae* fam. nov. (F3)**

*Krinioviventaceae* (Kri.ni.o.vi.ven.ta.ce’ae. N.L. masc./fem. n. *Kriniovivens* type genus of the family; L. suff. -*aceae* ending to denote a family; N.L. fem. pl. n. *Krinioviventaceaee* the *Kriniovivens* family).

The family *Krinioviventaceae* at present contains three genera *Kriniovivens* gen. nov., *Fontimicrobium* gen. nov., *Ca*. Fontivivens gen. nov. and *Hydrothermomicrobium* gen. nov.

Type genus: *Kriniovivens*.

***Kriniovivens* gen. nov. (G7)**

*Kriniovivens* (Kri.ni.o.vi’vens. Gr. n. *kríni* spring, fountain; L. pres. part. *vivens* living; N.L. masc./fem. n. *Kriniovivens* living in the spring).

Type species: *Kriniovivens* *secundus****.***

***Ca.* Kriniovivens primus sp. nov. (S11)**

*Kriniovivens primus* (pri’mus. L. masc. adj. *primus* first, to represent the first genome bin from of the genus).

Type material: Zod_Metabat.326 (GCA_016928635.1), obtained from the metagenome assembly of a sample from Zodletone Spring, Anadarko, USA.

***Kriniovivens secundus* sp. nov. (S12)**

*Kriniovivens secundus* (se.cun’dus. L. masc. adj. *secundus* second, to represent the second genome bin of the genus).

Type material: Zod_Metabat.251 (GCA_016929295.1), obtained from the metagenome assembly of a sample from Zodletone Spring, Anadarko, USA.

***Kriniovivens tertium* sp. nov. (S13)**

*Kriniovivens tertium* (ter’ti.um L. neut. adj. *tertium* third, to represent the first genome bin from of the genus).

Type material: Zod_Metabat.31 (GCA_016928775.1), obtained from the metagenome assembly of a sample from Zodletone Spring, Anadarko, USA.

***Fontimicrobium* gen. nov. (G8)**

*Fontimicrobium* (Fon.ti.mi.cro’bi.um. L. masc. n. *fons* (gen. *fontis*) a spring; N.L. neut. n. *microbium* a microbe; N.L. neut. n. *Fontimicrobium* a microbe from a spring).

Type species: *Fontimicrobium primus*.

***Fontimicrobium secundus* sp. nov. (S14)**

*Fontimicrobium secundus* (se.cun’dus. L. masc. adj. *secundus* second, to represent the second genome bin of the genus).

Type material: SRBZ_201705_bins_74 (JAPDQV000000000), obtained from the metagenome assembly of a sample from Zodletone Spring, Anadarko, USA.

***Fontimicrobium tertium* sp. nov. (S15)**

*Fontimicrobium tertium* (ter’ti.um. L. neut. adj. *tertium* third, to represent the first genome bin from of the genus).

Type material: DPZ2_202101_bins_149 (JAPDPN000000000), obtained from the metagenome assembly of a sample of hot spring located at Tengchong, PR China.

***Fontimicrobium primus* sp. nov. (S16)**

*Fontimicrobium primus* (pri’mus. L. masc. adj. *primus* first, to represent the first genome bin from of the genus).

Type material: metabat2.451 (GCA_003818595.1), obtained from the metagenome assembly of a sample from Cottonwood Lake study area, North Dakota, USA.

***Ca.* Fontivivens gen. nov. (G9)**

*Fontivivens* (Fon.ti.vi’vens. L. masc. n. *fons* (gen. *fontis*) a spring; L. pres. part. *vivens* living; N.L. masc./fem. n. *Fontivivens* living in the spring).

Type species: *Ca. Fontivivens zodletonensis*

***Ca.* Fontivivens zodletonensis sp. nov. (S17)**

*Fontivivens zodletonensis* (zod.le.ton.en’sis. N.L. masc/fem. adj. *zodletonensis,* pertaining to Zodletone Spring, Oklahoma, USA).

Type material: Zod_Metabat.863 (GCA_016934825.1), obtained from the metagenome assembly of a sample collected from Zodletone Spring located in Oklahoma, USA.

***Hydrothermomicrobium* gen. nov. (G10)**

*Hydrothermomicrobium* (Hy.dro.ther.mo.mi.cro’bi.um. Gr. neut n. *hydôr* water; Gr. masc. adj. *thermos* heat; N.L. neut. n. *microbium* a microbe; N.L. neut. n. *Hydrothermomicrobium* a microbe from hot water environment).

Type species: *Ca.* Hydrothermomicrobium *secundum*.

***Ca.* Hydrothermomicrobium primum sp. nov. (S18)**

*Hydrothermomicrobium primum* (pri’mum L. neut. adj. *primum* first, to represent the first genome bin from of the genus).

Type material: OTbin.62 (GCA_011682565.1), obtained from the metagenome assembly of a sample from a hydrothermal vent in East Pacific Rise of Pacific Ocean).

***Hydrothermomicrobium secundum* sp. nov. (S19)**

*Hydrothermomicrobium secundum* (se.cun’dum L. neut. adj. *secundum* second, to represent the second genome bin of the genus).

Type material: M_MetaBat.126 (GCA_013152225.1), obtained from the metagenome assembly of a sample of Okinawa hydrothermal vent).

***Sediminimicrobiaceae* fam. nov. (F4)**

*Sediminimicrobiaceae* (Se.di.mi.ni.mi.cro.bi.a.ce’ae. N.L. neut. n. *Sediminimicrobium* type genus of the family; L. suff. -*aceae* ending to denote a family; N.L. fem. pl. n. *Sediminimicrobiaceae* the *Sediminimicrobium* family).

The description is same as that of the genus *Sediminimicrobium*.

Type genus: *Sediminimicrobium*.

***Sediminimicrobium* gen. nov. (G11)**

*Sediminimicrobium* (Se.di.mi.ni.mi.cro’bi.um. L. neut n. *sedimen -nis* sediment; N.L. neut. n. *microbium* a microbe; N.L. neut. n. *Sediminimicrobium* a microbe from sediment).

Type species: *Sediminimicrobium riflense*.

***Sediminimicrobium riflense* sp. nov. (S20)**

*Sediminimicrobium riflense* (rifl.en’se. N.L. neut. adj. *riflense* referring to Rifle).

Type material: RBG_16_48_16 (GCA_001771235.1), obtained from the metagenome assembly of a sample from a well of Rifle background sediment).

***Residuimicrobiaceae* fam. nov. (F5)**

Residuimicrobiaceae (Re.si.du.i.mi.cro.bi.a.ce’ae. N.L. neut. n. *Residuimicrobium* type genus of the family; L. suff. -*aceae* ending to denote a family; N.L. fem. pl. n. *Residuimicrobiaceae* the *Residuimicrobium* family).

The family *Ca. Residuimicrobiaceae* contains two genera *Residuimicrobium* gen. nov., and *Anaerohabitans* gen. nov.

Type genus: *Residuimicrobium*.

***Residuimicrobium* gen. nov. (G12)**

*Residuimicrobium* (Re.si.du.i.mi.cro’bi.um. L. neut n. *residuum* residues, leftovers, tailings; N.L. neut. n. *microbium* a microbe; N.L. neut. n. *Residuimicrobium* a microbe from the residues or tailings).

Type species: *Residuimicrobium sunconrense*.

***Residuimicrobium suncorense* sp. nov. (S21)**

*Residuimicrobium suncorense* (sun.cor.en’se. N.L. neut. adj. *suncorense* of Suncor, referring to the source of genome from tailings pond of Suncor Energy, Canada)

Type material: UBA2214 (GCA_002327255.1), obtained from metagenome assembly of samples from Suncor tailings pond 6.

***Anaerohabitans* gen. nov. (G13)**

*Anaerohabitans* (A.nae.ro.ha′bi.tans. Gr. pref. *an-* not; Gr. masc. n. *aêr* (gen. *aeros*) air; L. pres. part. *habitans* inhabiting; N.L. masc. n. *Anaerohabitans* inhibiting a anaerobic environment).

Type species: *Anaerohabitans fermentens*.

***Anaerohabitans fermentans* sp. nov. (S22)**

*Anaerohabitans fermentans* (fer.men’tans. L. part. adj. *fermentans* fermenting, referring to the source of genome from anaerobic digestion of organic wastes).

Type material: AS27yjCOA_10 (GCA_012523655.1), obtained from the anaerobic digester metagenome).

***Oceaniaggregativiventaceae* fam. nov. (F6)**

*Oceaniaggregativiventaceae* (O.ce.a.ni.ag.gre.ga.ti.vi.ven.ta.ce’ae. N.L. masc./fem. n. *Oceaniaggregativivevns* type genus of the family; L. suff. -*aceae* ending to denote a family; N.L. fem. pl. n. *Oceaniaggregativiventaceae* the *Oceaniaggregativivevns* family).

The family *Oceaniaggregativiventaceae* contains two genera *Sulfursilanivivens* gen. nov. and *Oceaniaggregativevns* gen. nov.

Type genus: *Oceaniaggregativivevns*

***Sulfursilanivivens* gen. nov. (G14)**

*Sulfursilanivivens* (Sul.fur.si.la.ni.vi’vens. L. neut. n. sulfur, sulfur; L. masc. n. *silanus* a fountain, spring; L. pres. part. *vivens* living; N.L. masc. n. *Sulfursilanivivens* living in sulfide-rich spring).

Type species: *Sulfursilanivivens zodletonense*.

***Sulfursilanivivens zodletonense* sp. nov. (S23)**

*Sulfursilanivivens zodletonense* (zod.le.ton.en’se. N.L. neut. adj. *zodletonense*, referring to Zodletone Spring, Oklahoma).

Type material: Zod_Metabat.28 (GCA_016929055.1), obtained from the metagenome assembly of a sample of sulfide-rich spring in Oklahoma, USA.

***Oceaniaggregativivevns* gen. nov. (G15)**

*Oceaniaggregativivevns* (O.ce.a.ni.ag.gre.ga.ti.vi’vevns. L. masc. n. *oceanus*, the ocean; L. masc. perf. part. *aggregatus*, aggregated; N.L. masc./fem. n. *Oceaniaggregativivevns* living in the microbial mat on the seashore).

Type species: *Oceaniaggregativivevns primus*.

***Oceaniaggregativivevns primus* sp. nov. (S24)**

*Oceaniaggregativivevns primus* (pri’mus. L. neut. adj. *primus* first, to represent the first genome bin from of the genus).

Type material: Bin_228 (GCA_014728115.1) obtained from the metagenome assembly of a sample collected from Hameline Pool, located in Shark Bay, Australia.

***Oceaniaggregativivevns secundus* sp. nov. (S25)**

*Oceaniaggregativivevns secundus* (se.cun’dus. L. masc. adj. *secundus* second, to represent the second genome bin of the genus).

Type material: Bin_298 (GCA_014728085.1) obtained from the metagenome assembly of a sample collected from Hameline Pool, located in Shark Bay, Australia.

***Oleimicrobiales* ord. nov. (O3)**

Oleimicrobiales (O.le.i.mi.cro.bi.a’les. N.L. neut. n. *Oleimicrobium* type genus of the order; L. suff. -*ales* ending to denote an order; N.L. fem. pl. n. *Oleimicrobiales* the *Oleimicrobium* order).

The order *Oleimicrobiales* comprised of two families *Oleimicrobiaceae* fam. nov., and *Ca.* Geothermoviventaceae fam. nov.

Type genus: *Oleimicrobium*.

***Oleimicrobiaceae* fam. nov. (F7)**

*Oleimicrobiaceae* (O.le.i.mi.cro.bi.a.ce’ae. N.L. neut. n. *Oleimicrobium* type genus of the family; L. suff. -*aceae* ending to denote a family; N.L. fem. pl. n. *Oleimicrobiaceae* the family of the genus *Oleimicrobium*).

The description is same as that of the genus *Oleimicrobium*

Type genus: *Oleimicrobium*.

***Oleimicrobium* gen. nov. (G16)**

*Oleimicrobium* (O.le.i.mi.cro’bi.um. L. neut. n. *oleum* oil; N.L. neut. n. *microbium* a microbe; N.L. neut. n. *Oleimicrobium* a microbe living in oil.

Type species: *Oleimicrobium dongyingense.*

***Oleimicrobium calidum* sp. nov. (S26)**

calidum (ca’li.dum. L. neut. adj. *calidum* hot, referring to the source of the genome).

Type material: SZTDM2_201709_bins_136 (JAPDQW000000000), obtained from the metagenome assembly of a sample collected from hot spring in Tengchong, PR China.

***Oleimicrobium dongyingense* sp. nov. (S27)**

*dongyingense* (dong.ying.en’se N.L. neut. n. *dongyingense* of Dongying, referring to the source of the genome)

Type material: MAG-107 (GCA_014359355.1), obtained from metagenome assembly of samples from oil production facility in Dongying, PR China.

***Ca.* Geothermoviventaceae fam. nov. (F8)**

Geothermoviventaceae (Ge.o.ther.mo.vi.ven.ta.ce’ae. N.L. masc./fem. n. *Geothermovivens* type genus of the family; L. suff. -*aceae* ending to denote a family; N.L. fem. pl. n. *Geothermoviventaceae* the *Geothermovivens* family).

The family *Ca.* Geothermoviventaceae contains two genera *Ca.* Geothermovivens gen. nov., and *Zestofontihabitans* gen. nov.

Type genus: *Ca.* Geothermovivens.

***Zestofontihabitans* gen. nov. (G17)**

*Zestofontihabitans* (Zes.to.fon.ti.ha’bi.tans. Gr. masc. adj. *zestos* hot; L. masc. n. *fons* (gen. *fontis*) a spring; L. pres. part. *habitans* inhabiting; N.L. masc. n. *Zestofontihabitans* an inhabitant of hot spring).

Type species: *Zestofontihabitans yellowstonensis*.

***Zestofontihabitans yellowstonensis* sp. nov. (S28)**

*Zestofontihabitans yellowstonesis* (yel.low.ston.en’sis. N.L. masc./fem. adj. *yellowstonensis* referring to Yellowstone National Park)

Type material: SpSt-950 (GCA_011389025.1), obtained from the metagenome assembly of a sample collected from hot spring in Yellowstone National Park, USA.

***Zestofontihabitans tengchongensis* sp. nov. (S29)**

*Zestofontihabitans tengchongensis* (teng.chong.en’sis. N.L. masc./fem. adj. *tengchongensis* referring to Tengchong)

Type material: JZ2_201709_bins_23 (JAPDPT000000000), obtained from the metagenome assembly of a sample collected from hot spring in Tengchong, PR China.

***Ca.* Geothermovivens gen. nov. (G18)**

*Geothermovivens* (Ge.o.ther.mo.vi’vens. Gr. fem. n. *gê* the earth; Gr. masc. adj. *thermos* hot; L. pres. part. *vivens* living; N.L. masc./fem. n. *Geothermovivens* living in the hot earth).

Type species: *Geothermovivans pacificus.*

***Ca.* Geothermovivens pacificus sp. nov. (S30)**

*Geothermovivens pacificus* (pa.ci’fi.cus. L. masc. adj. *pacificus* peaceful, pertaining to the Pacific Ocean)

Type material: M_MaxBin.061 (GCA_013152765.1), obtained from the metagenome assembly of a hydrothermal vent in the East Pacific Rise of Pacific Ocean.

***Thermofontimicrobiales* ord. nov. (O4)**

*Thermofontimicrobiales* (Ther.mo.fon.ti.mi.cro.bi.a’les. N.L. neut. n. *Thermofontimicrobium* type genus of the order; L. suff. -*ales* ending to denote an order; N.L. fem. pl. n. *Thermofontimicrobiales* the *Thermofontimicrobium* order).

The order *Thermofontimicrobiales* comprised of four families *Thermofontimicrobiaceae* fam. nov., *Kaytiofontadaptataceae* fam. nov., *Ca.* Thiofontviventaceae fam. nov. and *Sulfurfontimicrobiaceae* fam. nov.

Type genus: *Thermofontimicrobium*.

***Thermofontimicrobiaceae* fam. nov. (F9)**

*Thermofontimicrobiaceae* (Ther.mo.fon.ti.mi.cro.bi.a.ce’ae. N.L. masc./fem. n. *Thermofontimicrobium* type genus of the family; L. suff. -*aceae* ending to denote a family; N.L. fem. pl. n. *Thermofontimicrobiaceae* the *Thermofontimicrobium* family).

The description is same as that of the genus *Thermofontimicrobium*.

Type genus: *Thermofontimicrobium*.

***Thermofontimicrobium* gen. nov. (G19)**

*Thermofontimicrobium* (Ther.mo.fon.ti.mi.cro’bi.um. Gr. masc. adj. *thermos* hot; L. masc. n. *fons*, a spring; N.L. neut. n. *microbium* a microbe; N.L. neut. n. Thermosalimicrobium a microbe from hot spring).

Type species: *Thermofontimicrobium primus*.

***Thermofontimicrobium primus* sp. nov. (S31)**

*Thermofontimicrobium primus* (pri’mus L. neut. adj. *primus* first, to represent the first genome bin from of the genus).

Type material: JZ4_201709_bins_129 (JAPDQG000000000), obtained from the of a sample collected from hot spring in Tengchong, PR China.

***Thermofontimicrobium secundus* sp. nov. (S32)**

*Thermofontimicrobium secundus* (se.cun’dus L. neut. adj. *secundus* second, to represent the second genome bin of the genus).

Type material: JZ4_201803_bins_154 (JAPDQM000000000), obtained from the of a sample collected from hot spring in Tengchong, PR China.

**Kaytiofontadaptataceae fam. nov. (F10)**

Kaytiofontadaptataceae (Kay.ti.o.font.ad.ap.ta.ta.ce’ae. Gr. adj. *kayti* hot; L. masc. n. *fons* (gen. *fontis*) spring; L. suff. -*aceae* ending to denote a family; N.L. fem. pl. n. *Kaytiofontadaptataceae* the *Kaytiofontadaptatus* family).

adapted to (living) in hot spring)

The family *Kaytiofontadaptataceae* at present contains four genera *Kaytiofontadaptatus* gen. nov. and *Ca.* Nigeripontimicrobium gen. nov.

Type genus: *Kaytiofontadaptatus*

***Kaytiofontadaptatus* gen. nov. (G20)**

*Kaytiofontadaptatus* (Kay.ti.o.font.ad.ap.ta.tus. Gr. adj. *kayti* hot; L. masc. n. *fons* (gen. *fontis*) spring; L. masc. perf. part. *adaptatus* adapted to; N.L. masc. n. *Kaytiofontadaptatus* adapted to (living) in hot spring).

Type species: *Kaytiofontadaptatus thermophilus*.

***Kaytiofontadaptatus thermophilus* sp. nov. (S33)**

*Kaytiofontadaptatus thermophilus* (ther.mo’phi.lus. Gr. fem. adj. *thermê* heat; N.L. masc. adj. suff. -*philus* friend, loving; N.L. masc. adj. *thermophilus* heat-loving, reflecting the source of the genome bin from a hot spring sediment).

Type material: SpSt-1156 (GCA_011046315.1), obtained from the metagenome assembly of a hot spring sediment sample collated from California, USA.

***Ca.* Nigeripontimicrobium gen. nov. (G21)**

*Nigeripontimicrobium* (Ni.ger.i.pon.ti.mi.cro’bi.um. L. masc. adj. *niger* black; N.L. masc. n. *pontus (*gen. *pontï*) the sea; from Gr. *póntus* sea; N.L. neut. n. *microbium* a microbe; N.L. neut. n. *Nigeripontimicrobium* a microbe from black sea).

Type species: *Ca.* Nigeripontimicrobium bulgariaensis.

***Ca.* Nigeripontimicrobium bulgaricum sp. nov. (S34)**

*Nigeripontimicrobium bulgaricum* (bul.ga’ri.cum. N.L. neut. adj. *bulgaricum* of Bulgaria, referring to the source of the genome).

Type material: BS750m-G10 (GCA_016783825.1), obtained from the metagenome assembly of a seawatert sample collated from Black Sea, Bulgaria.

***Ca.* Thiofontviventaceae fam. nov. (F11)**

Thiofontviventaceae (Thi.o.font.vi.ven.ta.ce’ae. N.L. masc. n. *Thiofontadaptatus* type genus of the family; L. suff. -*aceae* ending to denote a family; N.L. fem. pl. n. *Thiofontviventaceae* the *Thiofontadaptatus* family).

The family *Ca.* Thiofontviventaceae contains three genera, *Ca.* Thiofontadaptatus gen. nov., *Ca.* Mariaggregatimicrobium gen. nov. and *Ca.* Thiofontivivens gen. nov.

Type genus: *Ca.* Thiofontadaptatus.

***Ca.* Mariaggregatimicrobium gen. nov. (G22)**

Mariaggregatimicrobium (Ma.ri.ag.gre.ga.ti.mi.cro’bi.um. L. neut. n. *mare*, the sea; L. inf. v. *aggregare*, to come together, aggregate; N.L. neut. n. *microbium* a microbe; N.L. neut. n. *Mariaggregatimicrobium* a microbe from microbial mat on the seashore).

Type species: *Ca.* Mariaggregatimicrobium hamelinensis.

***Ca.* Mariaggregatimicrobium hamelinensis sp. nov. (S35)**

*Mariaggregatimicrobium hamelinensis* (ha.me.lin.en’sis. N.L. masc./fem. adj. *hamelinensis*, pertaining to Hamelin Pool, Australia, where the sample collected).

Type material: Bin_079 (GCA_014729735.1), obtained from the metagenome assembly of a sample collected from Hamelin Pool, Australia.

***Ca.* Thiofontivivens** **gen. nov.** **(G23)**

*Thiofontivivens* (Thi.o.fon.ti.vi’vens. Gr. neut. n. *theîon*, sulfur; L. masc. n. *fons* (gen. *fontis*) a spring; L. pres. part. *vivens* living; N.L. masc./fem. n. *Thiofontivivens* living in the sulfide-rich spring).

Type species: *Ca.* Thiofontivivens primus.

***Ca.* Thiofontivivens primus** **sp.** **nov. (S36)**

*Thiofontivivens primus* (pri’mus. L. masc. adj. *primus* first, to represent the first genome bin from of the genus).

Type material: Zod_Metabat.1197 (GCA_016931935.1), obtained from the metagenome assembly of a sample from Zodletone Spring, Anadarko, USA.

***Ca.* Thiofontadaptatus gen. nov. (G24)**

*Thiofontadaptatus* (Thi.o.font.ad.ap.ta’tus. Gr. neut. n. *theîon*, sulfur; L. masc. n. *fons* (gen. *fontis*) a spring; L. masc. perf. part. *adaptatus*, adapted to; N.L. masc. n. *Thiofontadaptatus* adapted to living in sulfide-rich spring).

Type species: *Ca.* Thiofontadaptatus primus

***Ca.* Thiofontadaptatus primus** **sp.** **nov.** **(S37)**

*Thiofontivivens primus* (pri’mus. L. masc. adj. *primus* first, to represent the first genome bin from of the genus).

Type material: Zod_Metabat.605 (GCA_016926625.1), obtained from the metagenome assembly of a sample from Zodletone Spring, Anadarko, USA.

***Sulfurfontimicrobiaceae* fam. nov. (F12)**

*Sulfurfontimicrobiaceae* (Sul.fur.fon.ti.mi.cro.bi.a.ce’ae. L. neut. n. sulfur, sulfur (S); L. masc. n. *fons* (gen. *fontis*) a spring; L. suff. -*aceae* ending to denote a family; N.L. fem. pl. n. *Sulfurfontimicrobiaceae* the *Sulfurfontimicrobium* family).

The description is same as that of the genus *Sulfurfontimicrobium*

***Sulfurfontimicrobium* gen. nov.** **(G25)**

*Sulfurfontimicrobium* (Sul.fur.fon.ti.mi.cro.bi’um. L. neut. n. sulfur, sulfur (S); L. masc. n. *fons* (gen. *fontis*) a spring; N.L. neut. n. *microbium* a microbe; N.L. neut. n. a microbe from sulfide-rich spring).

Type species: *Sulfurfontimicrobium sediment.*

***Sulfurfontimicrobium sedimenti* sp. nov. (S38)**

*Sulfurfontimicrobium sedimenti* (se.di.men’ti. L. gen. neut. n. *sedimenti*, from the sediment, pertaining to the source of the MAGs)

Type material: Zod_Metabat.436 (GCA_016927835.1), obtained from the metagenome assembly of a sediment sample from Zodletone Spring, Anadarko, USA.

***Oceanimicrobiales* ord. nov. (O5)**

*Oceanimicrobiales* (O.ce.a.ni.mi.cro.bi.a’les. N.L. neut. n. *Oceanimicrobium* type genus of the order; L. suff. -*ales* ending to denote an order; N.L. fem. pl. n. *Oceanimicrobiales* the *Oceanimicrobium* order).

The order *Ca. Oceanimicrobiales* comprised of two families *Oceanimicrobiaceae* fam. nov. and *Ca.* Mariniviventaceae fam. nov., which are all related to different Marine sediments.

Type genus: *Oceanimicrobium*.

***Oceanimicrobiaceae*** **fam. nov. (F13)**

*Oceanimicrobiaceae* (O.ce.a.ni.mi.cro'bi.a.ce.ae. N.L. neut. n. *Oceanimicrobium* type genus of the family; L. suff. -*aceae* ending to denote a family; N.L. fem. pl. n. *Oceanimicrobiaceae* the *Oceanimicrobium* family).

***Oceanimicrobium* gen. nov. (G26)**

*Oceanimicrobium* (O.ce.a.ni.mi.cro’bi.um. L. masc. n. *oceanus*, the ocean; N.L. neut. n. *microbium* a microbe; N.L. neut. n. *Oceanimicrobium* a microbe from the ocean).

Type species: *Oceanimicrobium pacifum.*

***Oceanimicrobium pacificum* sp. nov. (S39)**

*Oceanimicrobium pacificum* (pa.ci’fi.cum. L. neut. adj. *pacificum* pertaining to the Pacific Ocean, the source of the genome).

Type material: N075bin58 (GCA_004356825.1), obtained from the metagenome assembly of a marine sediment sample from Pacific Ocean.

***Ca.* Mariniviventaceae fam. nov. (F14)**

*Mariniviventaceae* (Ma.ri.ni.vi.ven.ta.ce’ae. N.L. masc./fem. n. *Marinivivens* type genus of the family; L. suff. -*aceae* ending to denote a family; N.L. fem. pl. n. *Mariniviventaceae* the *Marinivivens* family).

The description is same as that of the genus *Ca.* Marinivivens

Type genus: *Ca.* Marinivivens.

***Ca.* Marinivivens gen. nov. (G27)**

*Marinivivens* (Ma.ri.ni.vi’vens. L. masc. adj. *marinus* of the sea, marine; L. pres. part. *vivens* living; N.L. masc./fem. n. *Marinivivens* living in the sea).

Type species: *Ca.* Marinivivens kutchensis.

***Ca.* Marinivivens kutchensis sp. nov. (S40)**

*Marinivivens kutchensis* (kutch.en’sis. N.L. masc. adj. *kutchensis* of Kutch, referring to the Gulf of Kutch, India).

Type material: KS4-K089 (GCA_011774205.1), obtained from the metagenome assembly of a marine sediment sample from Gulf of Kutch, India.

## SUPPLEMENTARY FIGURES

**
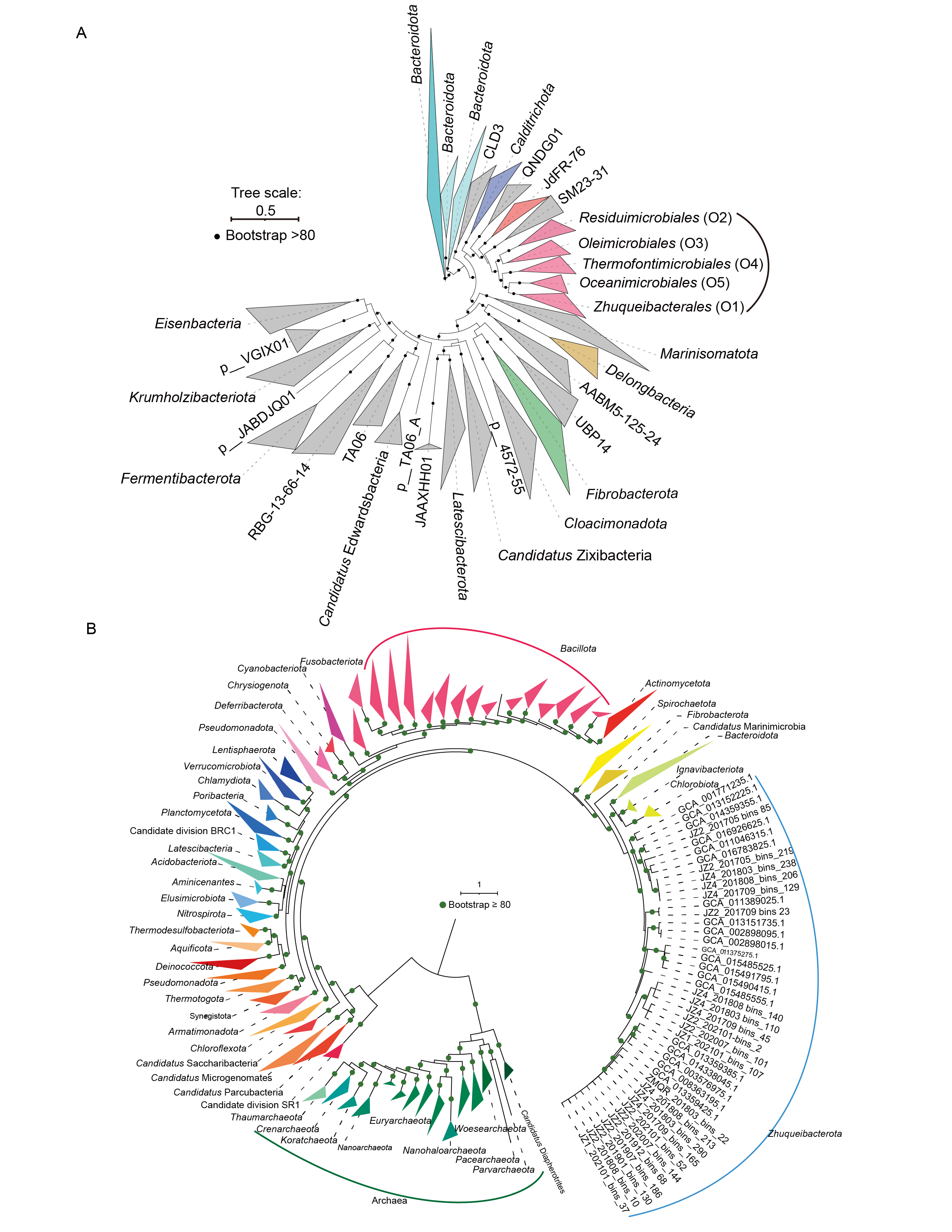
**

### **Figure S1. Phylogeny of *Zhuqueibacterota*.** A, The phylogenetic tree of FCB superphylum. The genome sequences of FCB superphylum were collected from GTDB_r207, and the Bac120 marker was identified using GTDB-Tk. B, The phylogenetic tree of 16S rRNA gene sequences. The 16S rRNA gene sequences were identified from the *Zhuqueibacterota* MAGs, while reference sequences were collected from the Usearch SINTAX reference database.

### **
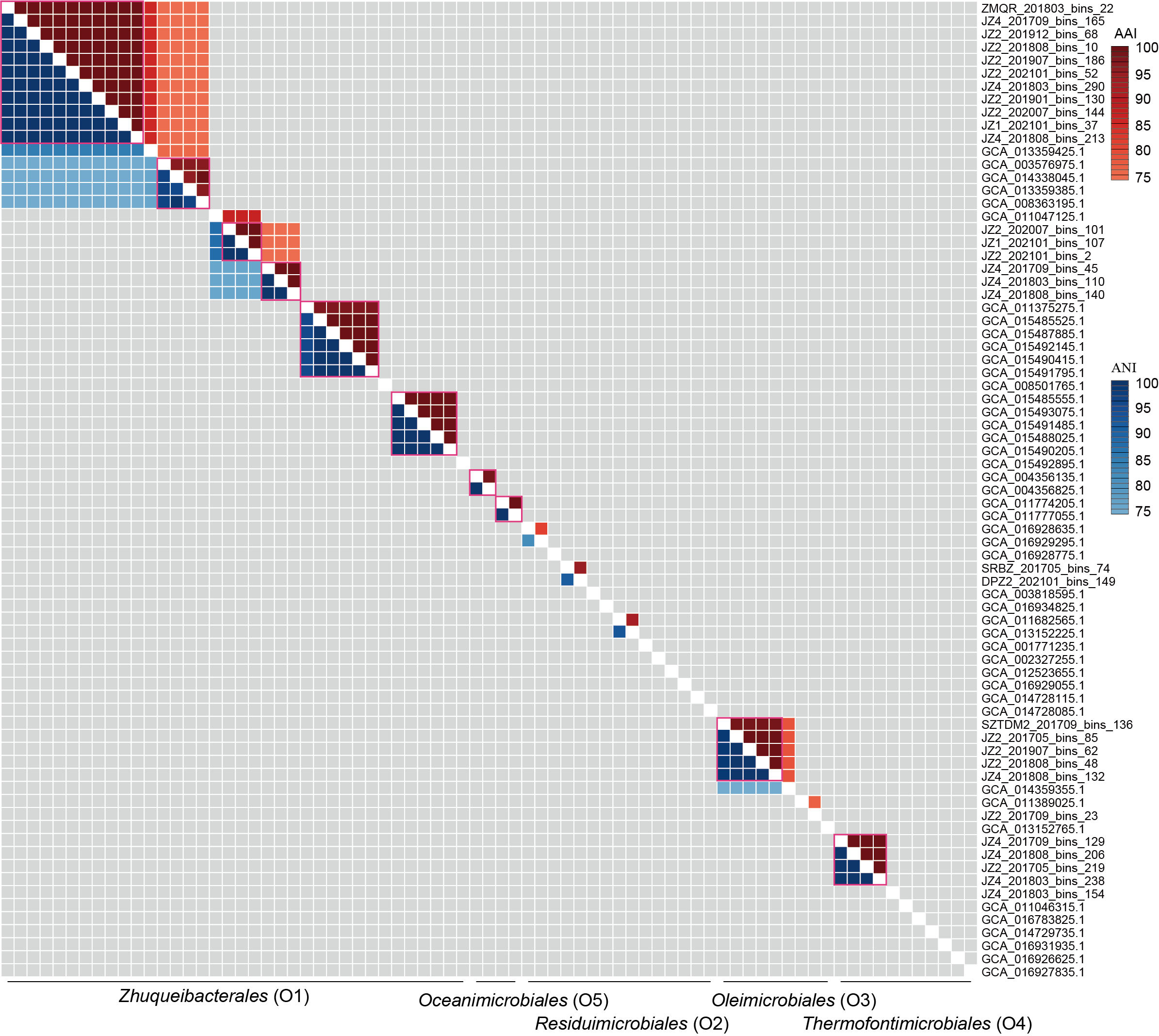
**

### **Figure S2. The ANI/AAI heatmap of all pairwise comparisons.** The upper triangle displays the AAI values, while the lower triangle displays the ANI values among genomes. Values below 75 are shaded in light gray.

### **
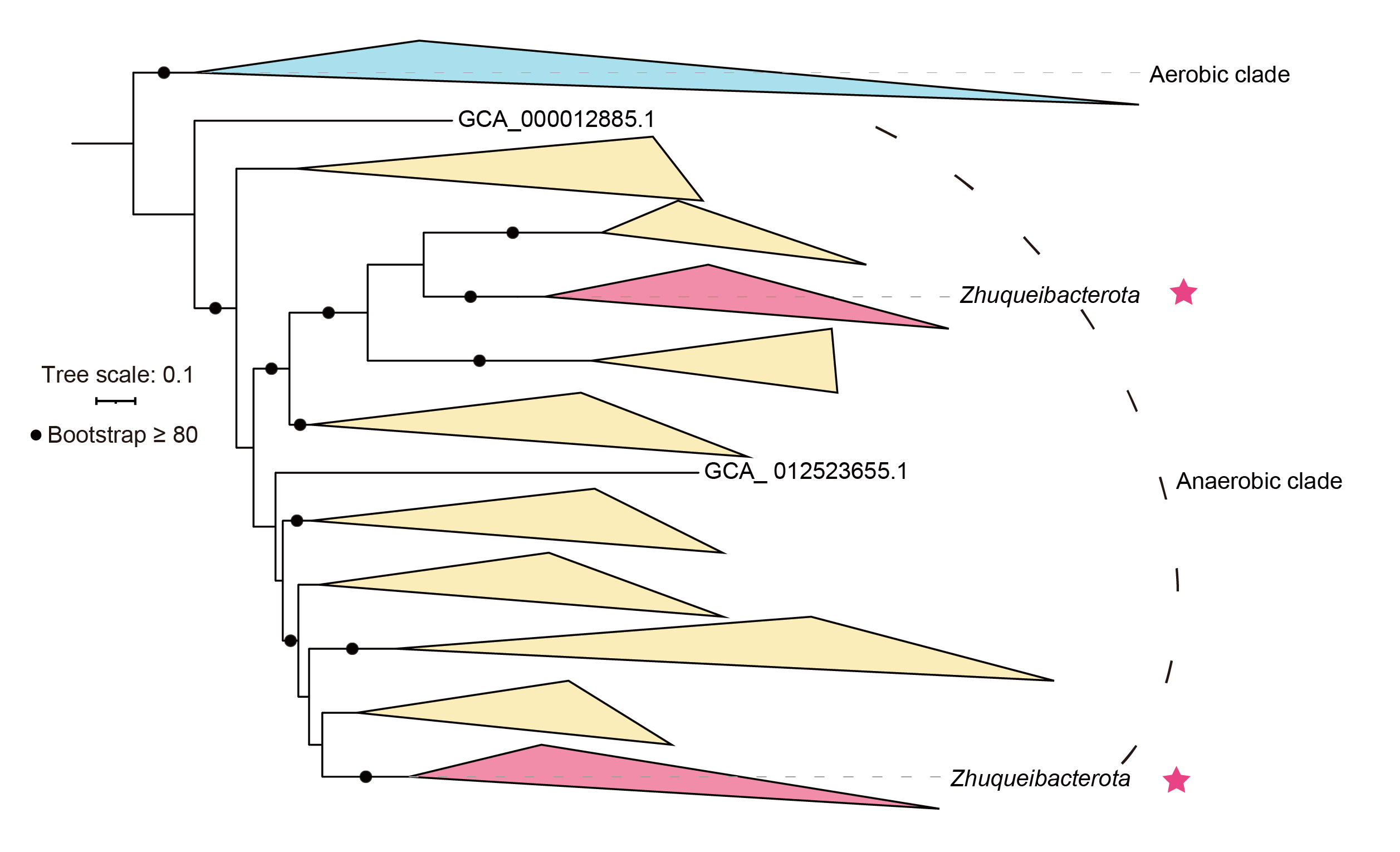
**

### **Figure S3. The phylogenetic tree based on concatenated alignment of Rnf complex genes.** The *rnf* gene cluster (*rnfABCDEG*) were identified, aligned, trimmed, and concatenated. The maximum likelihood tree was constructed using the IQ-Tree software. The aerobic and anaerobic traits of the species were ascertained by consulting the BacDive database.

### **
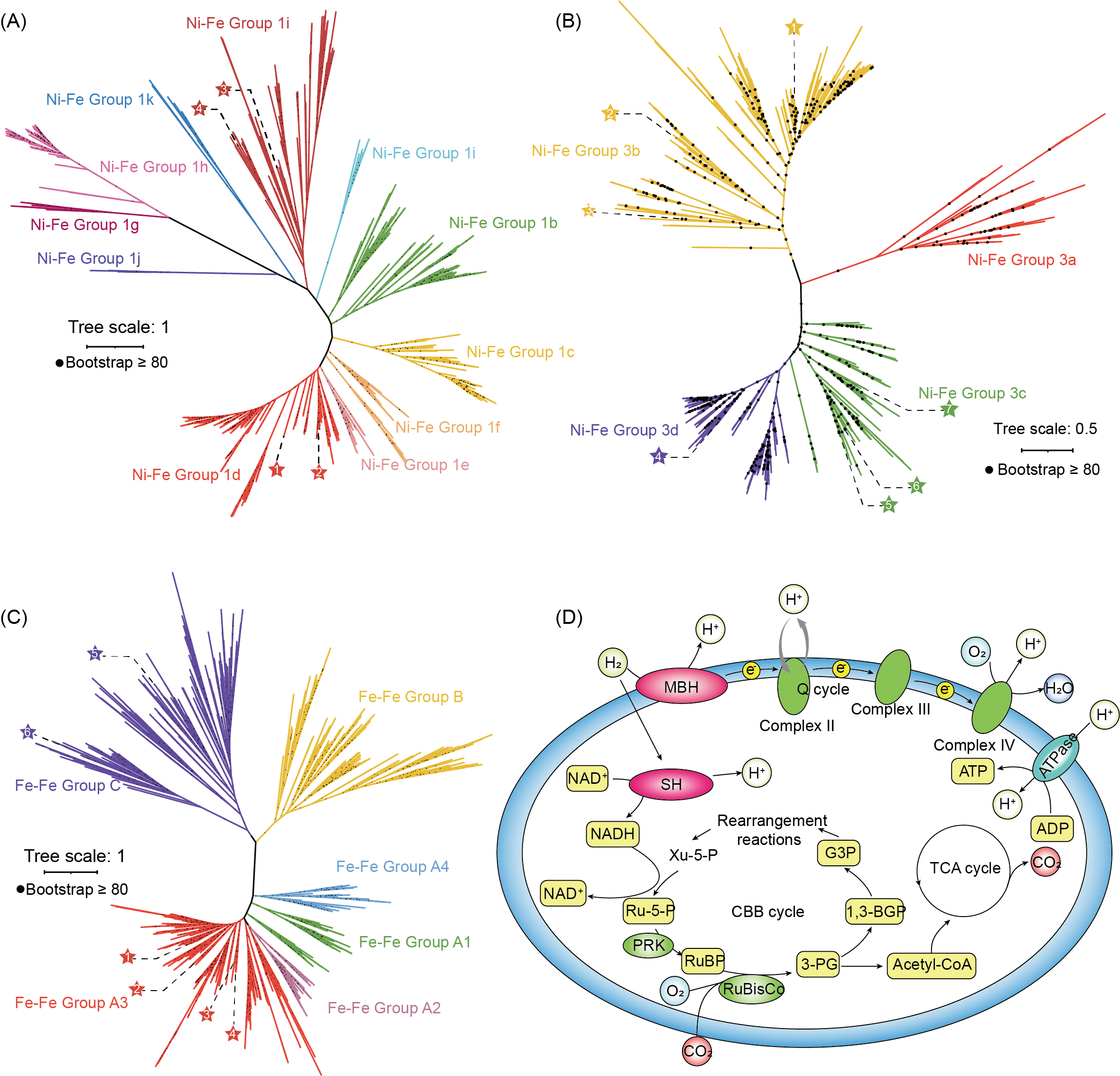
**

### **Figure** **S4. Phylogenetic trees and classification of hydrogenases and metabolic model of hydrogen-oxidizing bacteria.** Trees are colored by [NiFe]-hydrogenase subgroup and [FeFe]-hydrogenase group with black circles indicating well-supported nodes (bootstrap values ≥80). (A), Phylogenetic tree of [Ni-Fe] group 1 hydrogenase; (B), Phylogenetic tree [Ni-Fe] group 3; (C), Phylogenetic tree of [Fe-Fe] group; (D), Metabolic model depicting hydrogen oxidation, CBB cycle, TCA cycle, ATP/ADP generation, and electron transfer processes.

### **
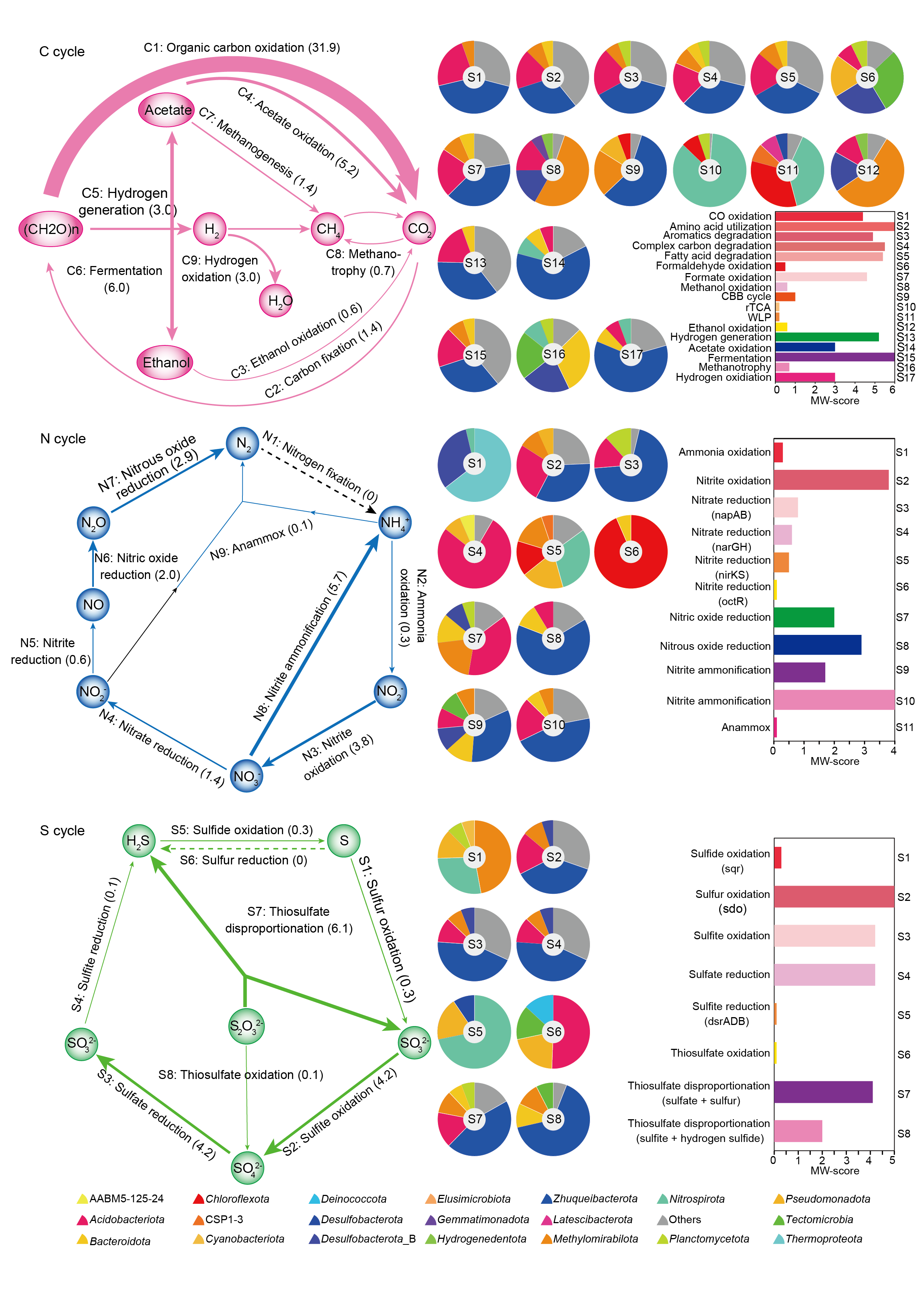
**

### **Figure S5. Microbial contribution to carbon, nitrogen, and sulfur cycling in hot spring.** Biogeochemical cycling diagrams profiling the community-level metabolic potential of C, N, and S cycling at the sampled site. The percentage of all genomes capable of conducting each metabolic step is listed next to each step. Arrow sizes represent the total relative abundance of genomes capable of carrying out the metabolic step. The pie charts depict the relative contribution of each phylum to a specific function within the microbial community. Species with relative contributions of less than 5% will be collectively labeled as "Others". The MW-score (metagenomic-weighted score) quantifies the potential metabolic interaction in the microbial community. The descriptions of function codes are listed in Table S8.

### **
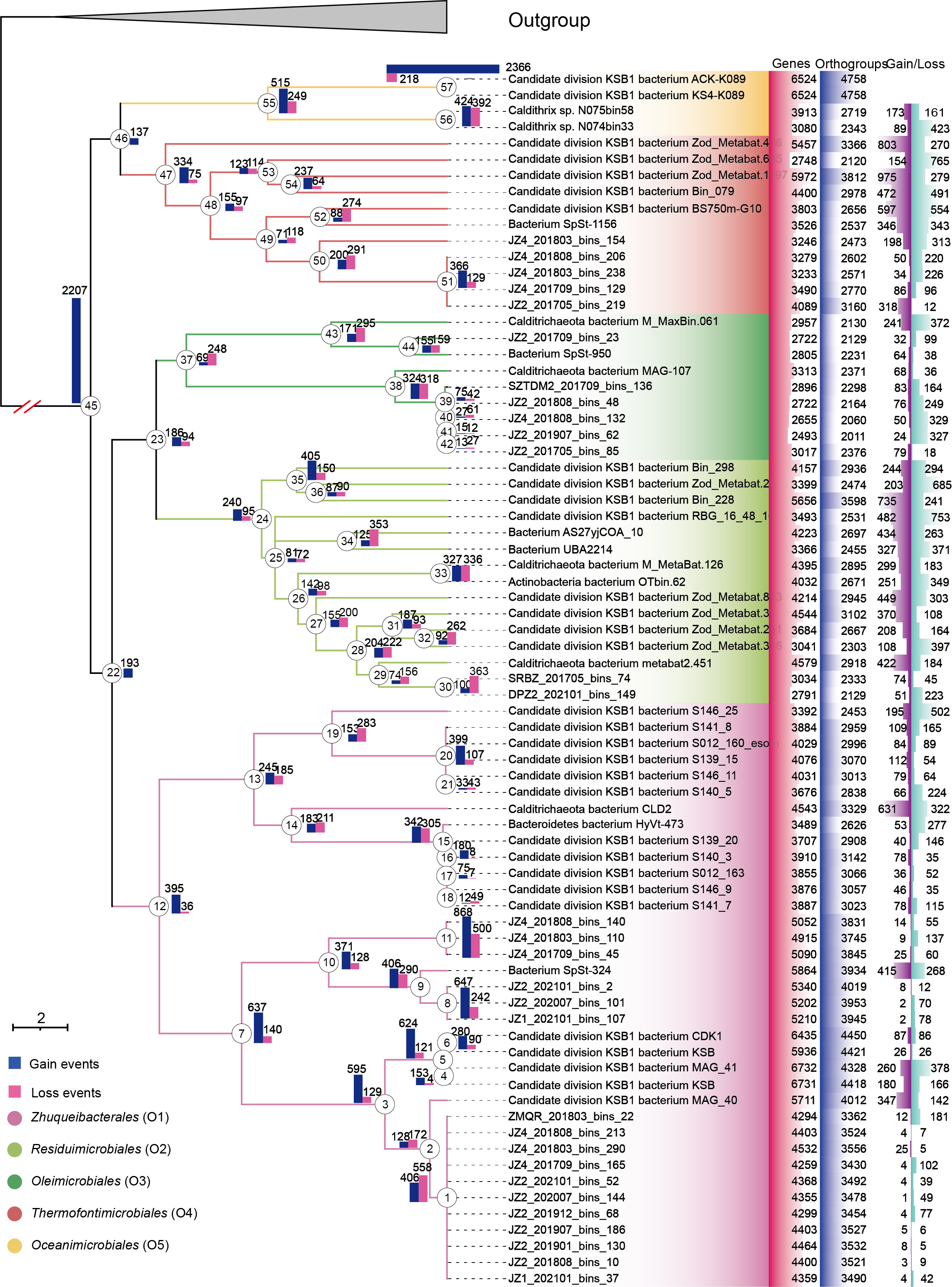
**

### **Figure S6. Ancestral genome content reconstruction using COUNT software.** The tree topology is derived from the Bayesian tree generated by MrBayes. Histograms adjacent to the internal node represent gene of gain (blue) and loss (pink) events. Bar plots on the right side of the tree display the number of genes, orthogroups, and gains/loss events for each genome analyzed in this study.

###
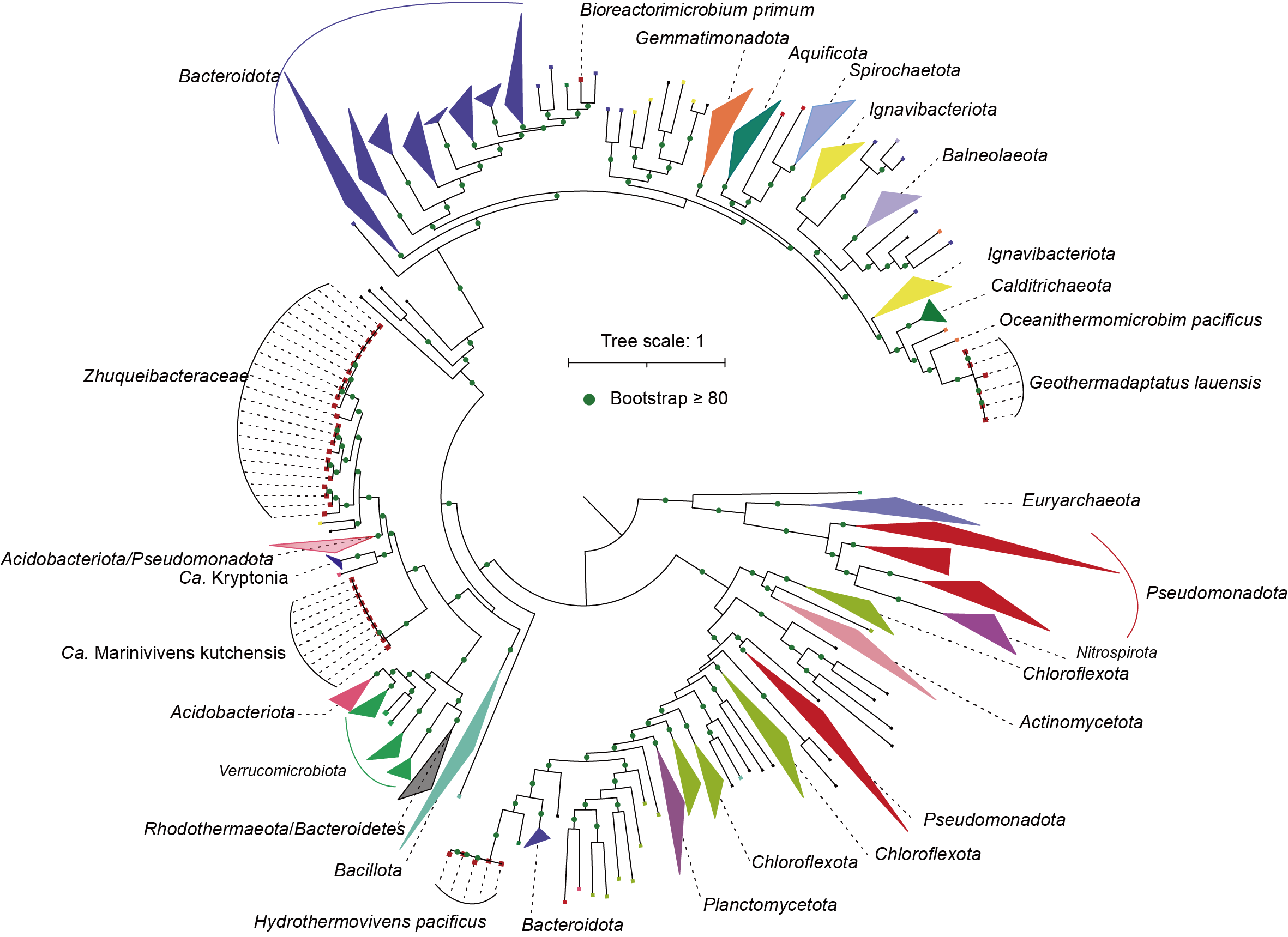


### **Figure S7. Phylogenetic tree of *nosZ* gene.** The *nosZ* reference sequence were obtained from a previous publication [37].

###
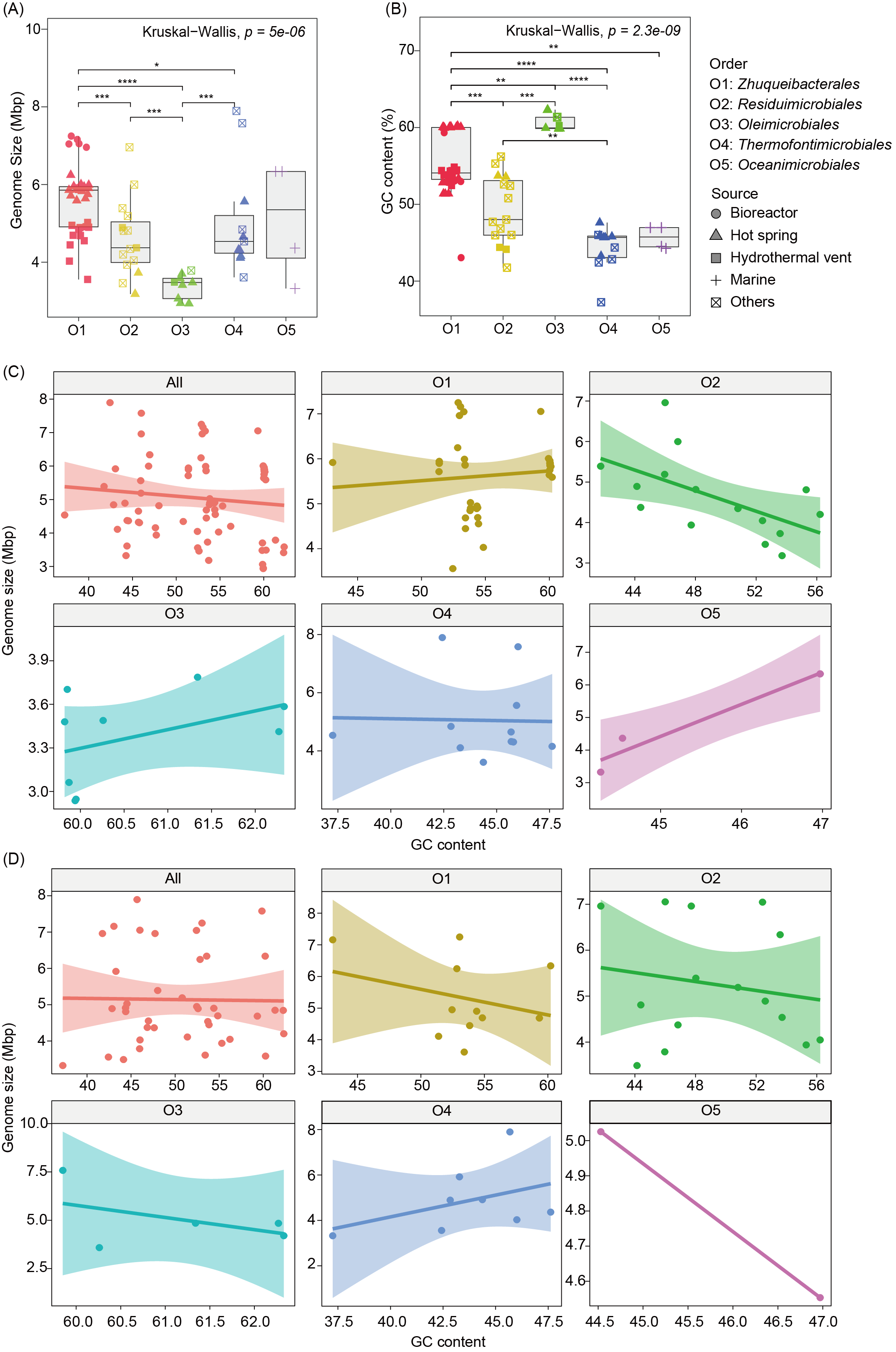


### **Figure S8. Variation and correlation of genome size and GC content across orders of *Zhuqueibacterota*.** (A), Genome size among different orders (Kruskal−Wallis, *p* = 5e-06); (B), GC content within each order (Kruskal−Wallis, *p = 2.3e-09*); (C), Correlation between the GC content and genome size among MAGs included in this study; (D), Correlation between GC content and genome size among species-level representative MAGs in this study.

###
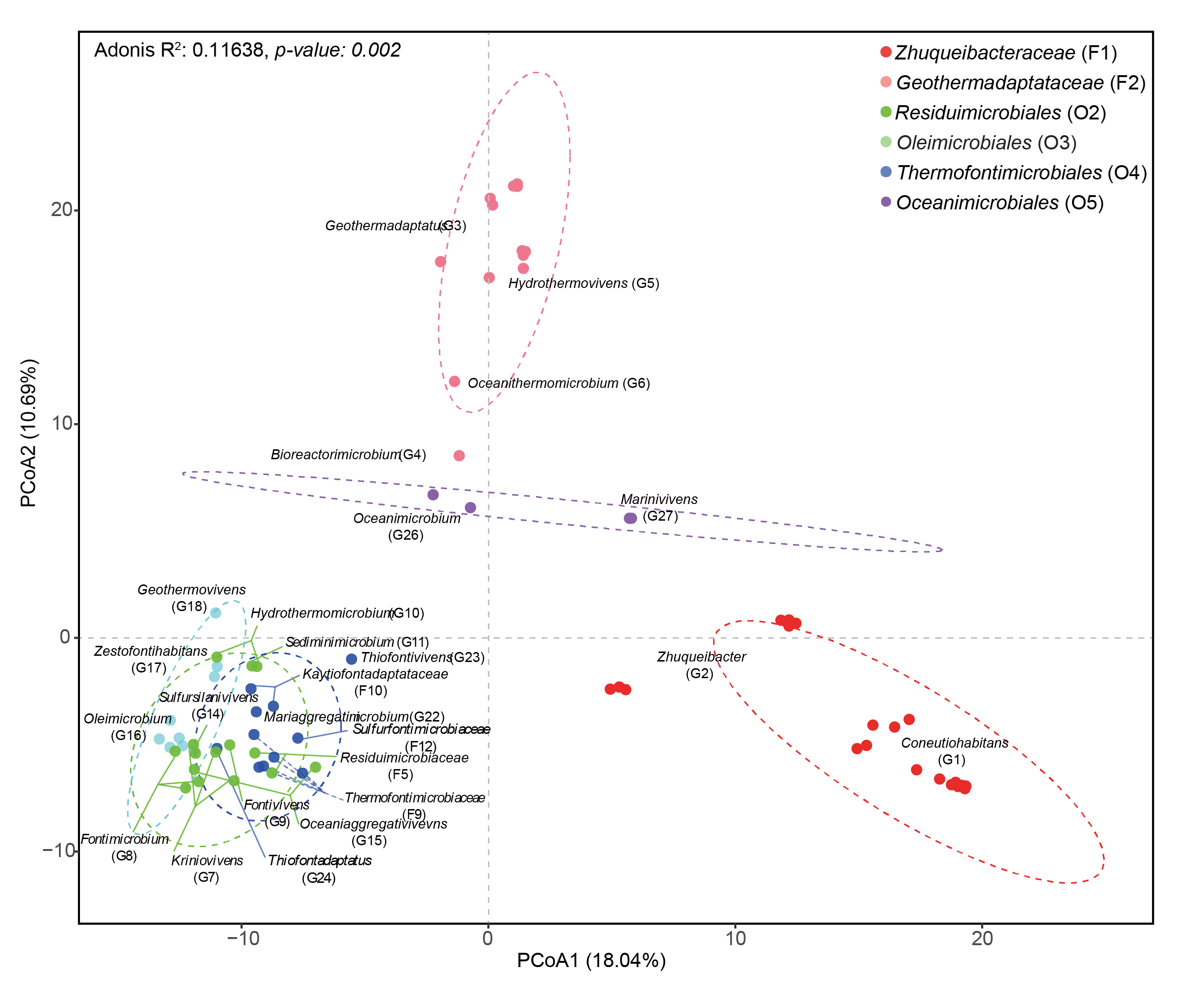


### **Figure S9. Plot of Principal Coordinates Analysis (PCoA) based on functional traits of *Zhuqueibacterota* MAGs.** The MAGs belonging to same order are labeled with the same color, while circles indicate the confidence intervals for MAGs from the same lineage.

## Reference

1. Jiao, Jian-Yu, Li Fu, Zheng-Shuang Hua, Lan Liu, Nimaichand Salam, Peng-Fei Liu, Ai-Ping Lv, et al. 2021. “Insight into the function and evolution of the Wood–Ljungdahl pathway in *Actinobacteria*.” *The ISME Journal* 15: 3005-3018. <https://doi.org/10.1038/s41396-021-00935-9>

2. Hua, Zheng-Shuang, Yu-Jiao Han, Lin-Xing Chen, Jun Liu, Min Hu, Sheng-Jin Li, Jia-Liang Kuang, Patrick S. G. Chain, Li-Nan Huang, Wen-Sheng Shu. 2015. “Ecological roles of dominant and rare prokaryotes in acid mine drainage revealed by metagenomics and metatranscriptomics.” *The ISME Journal* 9: 1280-1294. <https://doi.org/10.1038/ismej.2014.212>

3. Nurk, Sergey, Dmitry Meleshko, Anton Korobeynikov, Pavel A. Pevzner. 2017. “metaSPAdes: a new versatile metagenomic assembler.” *Genome Res* 27: 824-834. <https://doi.org/10.1101/gr.213959.116>

4. Kang, Dongwan D., Jeff Froula, Rob Egan, Zhong Wang. 2015. “MetaBAT, an efficient tool for accurately reconstructing single genomes from complex microbial communities.” *PeerJ* 3: e1165. <https://doi.org/10.7717/peerj.1165>

5. Parks, Donovan H., Michael Imelfort, Connor T. Skennerton, Philip. Hugenholtz, Gene W. Tyson. 2015. “CheckM: assessing the quality of microbial genomes recovered from isolates, single cells, and metagenomes.” *Genome Res* 25: 1043-1055. <https://doi.org/10.1101/gr.186072.114>

6. Hyatt, Doug, Gwo-Liang Chen, Philip F. LoCascio, Miriam L. Land, Frank W. Larimer, Loren J. Hauser. 2010. “Prodigal: prokaryotic gene recognition and translation initiation site identification.” *BMC Bioinformatics* 11: 119. <https://doi.org/10.1186/1471-2105-11-119>

7. Cantalapiedra, Carlos P., Ana Hernández-Plaza, Ivica Letunic, Peer Bork, Jaime Huerta-Cepas. 2021. “eggNOG-mapper v2: functional annotation, orthology assignments, and domain prediction at the metagenomic scale.” *Molecular Biology and Evolution* 38: 5825-5829. <https://doi.org/10.1093/molbev/msab293>

8. Moriya, Yuki, Masumi Itoh, Shujiro Okuda, Akiyasu C. Yoshizawa, Minoru Kanehisa. 2007. “KAAS: an automatic genome annotation and pathway reconstruction server.” *Nucleic Acids Research* 35: W182-W185. <https://doi.org/10.1093/nar/gkm321>

9. Aramaki, Takuya, Romain Blanc-Mathieu, Hisashi Endo, Koichi Ohkubo, Minoru Kanehisa, Susumu Goto, Hiroyuki Ogata. 2020. “KofamKOALA: KEGG ortholog assignment based on profile HMM and adaptive score threshold.” *Bioinformatics* 36: 2251-2252. <https://doi.org/10.1093/bioinformatics/btz859>

10. Laslett, Dean, Bjorn Canback. 2004. “ARAGORN, a program to detect tRNA genes and tmRNA genes in nucleotide sequences.” *Nucleic Acids Research* 32: 11-16. <https://doi.org/10.1093/nar/gkh152>

11. Zhang, Han, Tanner Yohe, Le Huang, Sarah Entwistle, Peizhi Wu, Zhenglu Yang, Peter K. Busk, Ying Xu, Yanbin Yin. 2018. “dbCAN2: a meta server for automated carbohydrate-active enzyme annotation.” *Nucleic Acids Research* 46: W95-W101. <https://doi.org/10.1093/nar/gky418>

12. Chaumeil, Pierre-Alain, Aaron J. Mussig, Philip Hugenholtz, Donovan H. Parks. 2020. “GTDB-Tk: a toolkit to classify genomes with the Genome Taxonomy Database.” *Bioinformatics* 36: 1925-1927. <https://doi.org/10.1093/bioinformatics/btz848>

13. Nguyen, Lam-Tung, Heiko A. Schmidt, Arndt von Haeseler, Bui Quang Minh. 2015. “IQ-TREE: a fast and effective stochastic algorithm for estimating maximum-likelihood phylogenies.” *Molecular Biology and Evolution* 32: 268-274. <https://doi.org/10.1093/molbev/msu300>

14. Kalyaanamoorthy, Subha, Bui Quang Minh, Thomas K. F. Wong, Arndt von Haeseler, Lars S. Jermiin. 2017. “ModelFinder: fast model selection for accurate phylogenetic estimates.” *Nature Methods* 14: 587-589. <https://doi.org/10.1038/nmeth.4285>

15. Edgar, Robert C. 2022. “Muscle5: high-accuracy alignment ensembles enable unbiased assessments of sequence homology and phylogeny.” *Nature Communications* 13: 6968. <https://doi.org/10.1038/s41467-022-34630-w>

16. Capella-Gutiérrez, Salvador, José M. Silla-Martínez, Toni Gabaldón. 2009. “trimAl: a tool for automated alignment trimming in large-scale phylogenetic analyses.” *Bioinformatics* 25: 1972-1973. <https://doi.org/10.1093/bioinformatics/btp348>

17. Jaffe, Alexander L., Cindy J. Castelle, Christopher L. Dupont, Jillian F. Banfield. 2019. “Lateral gene transfer shapes the distribution of RuBisCO among Candidate Phyla Radiation bacteria and DPANN archaea.” *Molecular Biology and Evolution* 36: 435-446. <https://doi.org/10.1093/molbev/msy234>

18. Edgar, Robert C. 2004. “MUSCLE: multiple sequence alignment with high accuracy and high throughput.” *Nucleic Acids Research* 32: 1792-1797. <https://doi.org/10.1093/nar/gkh340>

19. Greening, Chris, Ambarish Biswas, Carlo R. Carere, Colin J. Jackson, Matthew C. Taylor, Matthew B. Stott, Gregory M. Cook, Sergio E. Morales. 2016. “Genomic and metagenomic surveys of hydrogenase distribution indicate H_2_ is a widely utilised energy source for microbial growth and survival.” *The ISME Journal* 10: 761-777. <https://doi.org/10.1038/ismej.2015.153>

20. Matheus Carnevali, Paula B., Frederik Schulz, Cindy J. Castelle, Rose S. Kantor, Patrick M. Shih, Itai Sharon, Joanne M. Santini, et al. 2019. “Hydrogen-based metabolism as an ancestral trait in lineages sibling to the *Cyanobacteria*.” *Nature Communications* 10: 463. <https://doi.org/10.1038/s41467-018-08246-y>

21. Søndergaard, Dan, Christian N. S. Pedersen, Chris Greening. 2016. “HydDB: a web tool for hydrogenase classification and analysis.” *Scientific Reports* 6: 34212. <https://doi.org/10.1038/srep34212>

22. Biegel, Eva, Silke Schmidt, José M. González, Volker Müller. 2011. “Biochemistry, evolution and physiological function of the Rnf complex, a novel ion-motive electron transport complex in prokaryotes.” *Cellular and Molecular Life Sciences* 68: 613-634. <https://doi.org/10.1007/s00018-010-0555-8>

23. Letunic, Ivica, Peer Bork. 2019. “Interactive Tree Of Life (iTOL) v4: recent updates and new developments.” *Nucleic Acids Research* 47: W256-W259. <https://doi.org/10.1093/nar/gkz239>

24. Lagkouvardos, Ilias, Divya Joseph, Martin Kapfhammer, Sabahattin Giritli, Matthias Horn, Dirk Haller, Thomas Clavel. 2016. “IMNGS: A comprehensive open resource of processed 16S rRNA microbial profiles for ecology and diversity studies.” *Scientific Reports* 6: 33721. <https://doi.org/10.1038/srep33721>

25. Lee, I., Y. Ouk Kim, S. C. Park, J. Chun. 2016. “OrthoANI: an improved algorithm and software for calculating average nucleotide identity.” *Int J Syst Evol Microbiol* 66: 1100-1103. <https://doi.org/10.1099/ijsem.0.000760>

26. Kim, Dongwook, Sein Park, Jongsik Chun. 2021. “Introducing EzAAI: a pipeline for high throughput calculations of prokaryotic average amino acid identity.” *Journal of Microbiology* 59: 476-480. <https://doi.org/10.1007/s12275-021-1154-0>

27. Emms, David M., Steven Kelly. 2019. “OrthoFinder: phylogenetic orthology inference for comparative genomics.” *Genome Biology* 20: 238. <https://doi.org/10.1186/s13059-019-1832-y>

28. Csűös, Miklós. 2010. “Count: evolutionary analysis of phylogenetic profiles with parsimony and likelihood.” *Bioinformatics* 26: 1910-1912. <https://doi.org/10.1093/bioinformatics/btq315>

29. Huelsenbeck, John P., Fredrik Ronquist. 2001. “MRBAYES: Bayesian inference of phylogenetic trees.” *Bioinformatics* 17: 754-755. <https://doi.org/10.1093/bioinformatics/17.8.754>

30. Koutsovoulos, Georgios D., Solène Granjeon Noriot, Marc Bailly-Bechet, Etienne G. J. Danchin, Corinne Rancurel. 2022. “AvP: a software package for automatic phylogenetic detection of candidate horizontal gene transfers.” *PLOS Computational Biology* 18: e1010686. <https://doi.org/10.1371/journal.pcbi.1010686>

31. Suzek, Baris E., Yuqi Wang, Hongzhan Huang, Peter B. McGarvey, Cathy H. Wu, Consortium the UniProt. 2015. “UniRef clusters: a comprehensive and scalable alternative for improving sequence similarity searches.” *Bioinformatics* 31: 926-932. <https://doi.org/10.1093/bioinformatics/btu739>

32. Zhou, Zhichao, Patricia Q. Tran, Adam M. Breister, Yang Liu, Kristopher Kieft, Elise S. Cowley, Ulas Karaoz, Karthik Anantharaman. 2022. “METABOLIC: high-throughput profiling of microbial genomes for functional traits, metabolism, biogeochemistry, and community-scale functional networks.” *Microbiome* 10: 33. <https://doi.org/10.1186/s40168-021-01213-8>

33. Nielsen, Daniel A., Noah Fierer, Jemma L. Geoghegan, Michael R. Gillings, Vadim Gumerov, Joshua S. Madin, Lisa Moore, et al. 2021. “Aerobic bacteria and archaea tend to have larger and more versatile genomes.” *Oikos* 130: 501-511. <https://doi.org/10.1111/oik.07912>

34. Konstantinidis, Konstantinos T., James M. Tiedje. 2004. “Trends between gene content and genome size in prokaryotic species with larger genomes.” *Proceedings of the National Academy of Sciences* 101: 3160-3165. <https://doi.org/10.1073/pnas.0308653100>

35. Wu, Hao, Zhang Zhang, Songnian Hu, Jun Yu. 2012. “On the molecular mechanism of GC content variation among eubacterial genomes.” *Biology Direct* 7: 2. <https://doi.org/10.1186/1745-6150-7-2>

36. Chuckran, Peter F., Bruce A. Hungate, Egbert Schwartz, Paul Dijkstra. 2021. “Variation in genomic traits of microbial communities among ecosystems.” *FEMS Microbes* 2: xtab020. <https://doi.org/10.1093/femsmc/xtab020>

37. Jiao, Jian-Yu, Zheng-Han Lian, Meng-Meng Li, Nimaichand Salam, En-Min Zhou, Lan Liu, Hong Ming, et al. 2022. “Comparative genomic analysis of *Thermus* provides insights into the evolutionary history of an incomplete denitrification pathway.” *mLife* 1: 198-209. <https://doi.org/10.1002/mlf2.12009>
